# Transferrin conjugated pH/NIR-responsive black phosphorus nanoplatform: A novel multimodal approach for breast cancer theranostics

**DOI:** 10.1016/j.ijpx.2025.100364

**Published:** 2025-07-22

**Authors:** Soji Soman, Sanjay Kulkarni, Jeena John, Milan Paul, Krishnadas Nandakumar, Swati Biswas, Sajan D. George, Srinivas Mutalik

**Affiliations:** a Department of Pharmaceutics, Manipal College of Pharmaceutical Sciences, Manipal Academy of Higher Education, Manipal 576104, Karnataka, India; b Department of Pharmacology, Manipal College of Pharmaceutical Sciences, Manipal Academy of Higher Education, Manipal 576104, Karnataka, India; c Department of Pharmacy, Birla Institute of Technology & Science-Pilani, Hyderabad Campus, Medchal, Hyderabad 500078, Telangana State, India; d Centre for Applied Nanoscience, Manipal Institute of Applied Physics, Manipal Academy of Higher Education, Manipal 576102, Karnataka, India

**Keywords:** Nanotheranostic, Breast cancer, Phototherapy, Black phosphorous, 2D material

## Abstract

Cancer is one of the foremost causes of death, posing a significant challenge to global health. Breast cancer is predominant cancer type in women globally. Nanotheranostic platforms have emerged as a promising strategy for enhancing breast cancer therapy by integrating both diagnostic and therapeutic functionalities. Black phosphorous nanosheets (BPNSs) are nanotheranostic 2D materials that can adsorb a large number as well as quantity of therapeutic or theranostic agents due to their greater surface area. Herein, the authors developed an efficient pH and NIR-responsive nanotheranostic drug delivery platform for synergistic chemo/photothermal/photodynamic therapy of breast cancer. The exfoliated BPNSs were surface loaded with an anticancer drug, doxorubicin (DOX), functionalized with a photo-responsive polymer, polydopamine (PDA), and conjugated with a protein-based ligand, transferrin (TF). The prepared BP-based nanoformulation demonstrated excellent photothermal stability, improved targeting efficiency and desired therapeutic activity. A pH/NIR-responsive nature was observed during in vitro DOX release studies with negligible toxicity during hemolysis and CAM assay. Cell line studies on 4 T1 cells showed NIR-specific toxicity compared to non-irradiated groups with excellent ligand-mediated cellular uptake. Furthermore, an increase in the amount of necrotic and apoptotic cells in 4 T1 cells with elevated ROS levels were observed. 4 T1-tumour bearing BALB/c mice models administered with BP-based nanoformulation indicated an efficient reduction in tumour volume in NIR-irradiated groups and enhanced pharmacokinetic parameters. In vitro and in vivo studies demonstrated that the synergistic approach of merging chemotherapy with photothermal and photodynamic therapy exhibits remarkable antitumour effectiveness, excellent biocompatibility, and promising competence for clinical application.

## Graphical abstract

**Figure:**
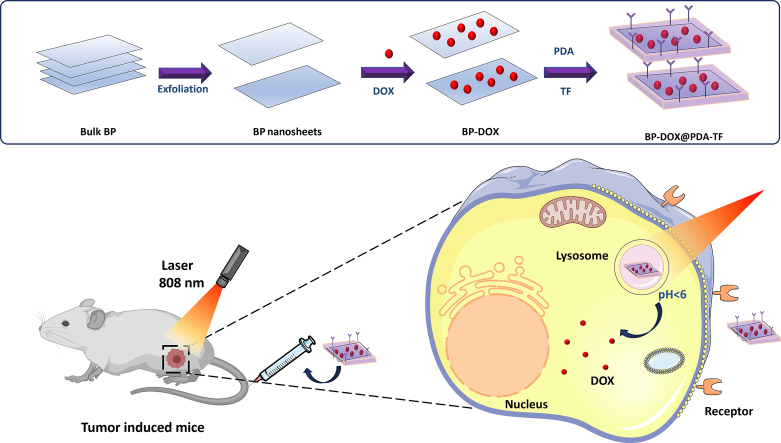

## 1 Introduction

Cancer is one of the most harmful diseases impacting humanity. In recent decades, significant efforts have been undertaken to combat against cancer (Hai et al., 2019). Cancer is the uncontrolled division of cells because of genetic impairment. Breast cancer is prevailing cancer type in women worldwide. The key targets involved in breast cancer are estrogen receptor alpha (ERα) and human epidermal growth factor receptor 2 (HER2/neu, encoded by ERBB2). A major subtype, triple-negative breast cancer (TNBC), is characterized by the lack of expression of estrogen receptor (ER), progesterone receptor (PR), and HER2 (ERBB2), and accounts for approximately 15 % of all breast cancer cases (Waks and Winer, 2019). Because of absence of specific molecular targets and the heterogeneity of the tumours, chemotherapy remains the primary treatment option for TNBC.

The common breast cancer treatment strategies include chemotherapy, hormone therapy and radiotherapy. In the early stages, surgery is the centrepiece of treatment. A major limitation related with these strategies is the lack of specificity. Over the past decades of research, nanomedicines made drastic improvement in cancer treatment, which is mostly concentrating on the fabrication and biomedical applications of technologies and nanomaterials at the nanoscale (Tang et al., 2017). Conventional therapy fails to target specifically the cancer cells, so they cause significant side effects which include organ damage. Chemotherapeutics may be rapidly removed from the systemic circulation as a result of their engulfment by macrophages. It shortens the drug's circulation time, rendering it ineffective against malignant cells. In addition, chemotherapeutic agents are typically hydrophobic in nature which causes reduced bioavailability (Sebastian, 2017; Sutradhar and Amin, 2014).

Recent studies have explored nanomedicine strategies aimed at reprogramming the tumour microenvironment. Macrophage-inherited exosomes have been shown to alleviate immunosuppression and promote immune-activated ferroptosis in tumours, offering a novel immunotherapeutic mechanism (Wang et al., 2023). Similarly, single-atom iron nanozymes with axially coordinated O–Fe–N₄ centres were reported to modulate macrophage epigenetics, thereby reshaping the immune landscape within tumour (Wang et al., 2024). These findings underscore the potential of smart nanoplatforms not only for direct tumour targeting but also for immune modulation. Nanotheranostics represents a highly promising method in personalized medicine by integrating both therapy and diagnosis into a single nanoplatform. This method allows for the detection of targets, non-invasive monitoring of drug distribution, evaluation of therapeutic activities, and optimization of individualized treatment, ultimately improving patient safety (Chen et al., 2017b; Liu et al., 2019a).

2D nanomaterials one of the nanotheranostic agents such as graphene-based materials, MXenes, and transition metal dichalcogenides that have gained wide consideration due to their unique optical as well as electronic properties. 2D nanomaterials offer promising applications in breast cancer for targeted drug delivery, imaging, and combined therapies (Mohammadpour and Majidzadeh-A, 2020; Parihar et al., 2025). 2D materials may have a reduced surface area for efficient loading of imaging or anti-tumour agents. Some materials elicit an immunological response and have hazardous potential. The loading of imaging agents into this system may result in inadequate absorption and substantial tissue toxicity (Gao et al., 2019; Kulkarni et al., 2024; Soman et al., 2024a, Soman et al., 2024b). However, these constraints can be circumvented by using two-dimensional materials of BP. Black phosphorous nanosheets (BPNSs) can adsorb a large number as well as quantity of therapeutic or theranostic agents due to their greater surface area. They do not cause an immunological response or have a hazardous potential since they break down in vivo to phosphates and phosphonates that are not toxic. BPNS itself has imaging ability, so it doesn't need any additional imaging agent. BPNSs display improved surface area, which allows efficient loading of chemotherapeutic agents (Qian et al., 2017; Soman et al., 2022). Moreover, it was reported that BPNSs are suitable nanocarriers capable of accommodating chemotherapeutic agents that are heavier than them, making it as a promising candidate for chemo-photothermal cancer treatment (Liu et al., 2019b). At near infrared (NIR) range, BPNSs exhibit excellent photothermal conversion feature, which makes them a suitable candidate for photothermal therapy (PTT). When BPNSs exposed to NIR radiation, it will create toxic singlet oxygen (^1^O_2_), so that it can be chosen as a photosensitive agent for photodynamic therapy (PDT) of cancer (Qian et al., 2017).

Doxorubicin (DOX) is an anthracycline derivative known for its strong anticancer effects across various tumour cells. It works by intercalating into DNA base pairs, disrupting transcription and replication, thereby hindering DNA and RNA synthesis. Additionally, DOX increases oxidative damage, leading to DNA breakage. However, its use in cancer treatment is limited by dose-related cardiotoxicity and myelosuppression (Rochette et al., 2015; Zhao and Zhang, 2017). One effective strategy to mitigate the adverse effects of antitumour drugs is to encapsulate them within a nanoplatform. The anticancer efficacy can be further improved by targeted internalization of nanoplatforms into or near tumour cells. This can be accomplished with targeting nanoparticles using ligand modification and functionalization(Song et al., 2013). Polydopamine (PDA), a photothermal agent formed through the oxidative self-polymerization of dopamine. PDA effectively absorbs NIR light, exhibiting excellent photothermal conversion properties. Additionally, PDA can enhance the biocompatibility of NPs and may significantly extend their circulation time when administered intravenously (Zhang et al., 2015).

Transferrin (TF) is a glycoprotein mostly generated in the liver. The primary function of TF is to transfer iron throughout the systemic circulation. TF binds to specific receptors that are overexpressed on breast tumour cells, enabling targeted drug delivery (Vandewalle et al., 1985). Soe and team created a DOX delivery method using TF-conjugated polymeric nanoparticles for the breast cancer cell therapy (Soe et al., 2019). TF-conjugated CQDs in combination with an antitumour medication DOX have been used to treat brain tumour (Li et al., 2016). A recent study successfully developed DOX loaded BP nanosheets for tumour-targeted drug delivery and photothermal therapy, demonstrating effective tumour ablation under NIR irradiation (Chen et al., 2017a). The current work designed a transferrin-conjugated BP-based nanoplatform co-loaded with doxorubicin and functionalized with PDA, offering active tumour targeting, pH/NIR-triggered release, and enhanced therapeutic synergy through chemo–photothermal–photodynamic modalities. PDA layer coating was used to cover the nanosheet surface, limiting DOX leakage in a physiological pH of 7.4 and demonstrating sustained-release behaviour at a lower pH of 5.0 while maintaining excellent NIR absorption. TF was chosen as the target ligand because the TF receptor is overexpressed in many cancers. In addition, our system enables real-time photothermal imaging and includes comprehensive in vitro and in vivo analyses, positioning it as a more robust and clinically relevant nanotheranostic candidate.

## 2 Materials and methods

### 2.1 Materials

Fluorescein 5-isothiocyanate, transferrin, trehalose, Triton X-100, Dopamine hydrochloride, and the dialysis membrane (MWCO 12 to 14 kDa) were purchased from Sigma–Aldrich, USA. Sodium dodecyl sulfate, and daunorubicin were purchased from TCI, Tokyo, Japan. Orthophosphoric acid (85 %), Tween 80, and ethylenediaminetetraacetic acid (EDTA) were purchased from Merck Life Sciences, Mumbai, India. Black phosphorous was procured from Jiangsu XFNANO Materials Tech Co. Ltd., China and Doxorubicin hydrochloride (HCl) was obtained as a gift sample from Neon Laboratories Ltd., Mumbai, India.

### 2.2 Preformulation studies of drug and bulk BP

Preformulation studies are critical for establishing a drug's physicochemical properties and compatibility with excipients. In this context, the following preformulation tests were performed on the drug DOX:

#### 2.2.1 Identification of drug by DSC

The thermal characteristic of drug was analyzed with differential scanning colorimetry (DSC 60 plus, Shimadzu). It detects thermal events such as melting and crystallization, providing information about the material's thermal properties. 8 mg of drug was taken and scanned at 0–250 °C range (10 °C/min). Nitrogen (N_2_) was purged at a rate of 40 mL/min to keep an inert atmosphere (Alves et al., 2022; Bandopadhyay et al., 2018).

#### 2.2.2 Identification of drug by FTIR

The FTIR-ATR technique involves passing an infrared beam through an ATR crystal that is in contact with the sample. Infrared light reflects internally and interacts with the sample surface, resulting in the absorption of wavelengths that correspond to the sample's molecular vibrations. This interaction generates an infrared spectrum, which is used to determine the sample's chemical composition. The drug and drug-carrier interaction were analyzed with FTIR spectrometer (Bruker Alpha II), scanned at a range of 4000 cm^−1^ to 500 cm^−1^ (Beraldo-Araújo et al., 2022).

#### 2.2.3 Characterization of bulk BP

Bulk BP (99.995 %) was purchased from XF-Nano (China) and stored in inert atmosphere. The collected BP was characterized in terms of SEM, XRD, Raman, EDS and AT-FTIR spectroscopy. SEM can be used to confirm the layered structure of BP. Raman spectroscopy as well as Raman spectroscopy were used to elucidate the crystalline structure of the BP material. EDS is essential for evaluating the chemical purity of the sample.

### 2.3 Synthesis of BPNSs from bulk BP and its characterization

The BPNSs was obtained with an improved liquid exfoliation using the obtained bulk BP. First, we grinded bulk BP in a mortar to get BP powder. 10 mg of BP powder was then added to 20 mL of *n*-methyl-2-pyrrolidone (NMP) and probe sonicated (Amplifier: 25 %, on/off cycle: 3 s/2 s). The ice water bath was used to avoid the high temperature effect of the system. The unexfoliated BP was separated by centrifugation at 2000 rpm for 10 min from the resulting brown solution. The supernatant containing exfoliated BPNSs was then collected by centrifugation at 14000 rpm for 30 mins and stored in 4 °C for further use. The exfoliated nanosheets were characterized by microscopically as well as spectroscopically (Yang et al., 2019).

#### 2.3.1 Characterization of BPNSs

The synthesized BPNSs were characterized using various techniques.

##### 2.3.1.1 Particle size, zeta potential and surface morphology

Size determines the average diameter of nanoparticles, which is critical for understanding their distribution and prospective applications. The zeta potential measures particle surface charge, which indicates stability in colloidal dispersions. The Polydispersity Index (PDI) measures the uniformity of particle sizes within a sample, with lower values indicating more uniform sizes. The synthesized BPNSs was characterized for particle size, PDI, and zeta potential using a Malvern Zetasizer (Nano ZS, Malvern Instruments Ltd., UK). The surface morphology of the nanoparticles was analyzed using scanning electron microscopy (SEM) (EVO MA18 Oxford EDS).

##### 2.3.1.2 Spectroscopic analysis

Several spectroscopic techniques were employed to analyze BPNSs. The FTIR spectrum was obtained using a Bruker Alpha II ATR-FTIR instrument, scanned over a range of 500–4000 cm^−1^, with baseline correction and normalization for transmittance. The crystalline structure of BPNSs was assessed using powder X-ray diffraction (PXRD) with an Miniflex 600 (5th gen) (Rigaku, Tokyo, Japan), complemented by Raman spectroscopy (B&W TEK, i-Raman Plus, Plainsboro, NJ, USA). Additionally, EDS (EVO MA18 Oxford EDS) was performed to determine the elemental composition of BPNSs. Attached to an electron microscope, EDS detects characteristic X-rays released by the sample to establish elemental composition. It performs qualitative and quantitative analyses of constituents in the sample, down to the microscale.

### 2.4 Formulation and characterization of BP-based nanoformulation

The collected BPNSs were loaded with a chemotherapeutic agent (DOX), functionalized with a biopolymer PDA and attached with a protein-based ligand, TF for precise localization of breast cancer cells. The optimized formulation was then characterized by various techniques.

#### 2.4.1 Loading of DOX into exfoliated BPNSs

To a solution of 1 mg/mL of DOX, 1 mg exfoliated BP was added and adjusted to pH 7.4 with NaOH. The BP-DOX solution was then vigorously stirred in dark for overnight and collected the DOX loaded BPNSs by centrifugation (15,000 rpm for 30 mins). The DOX loading efficiency of the prepared BP-DOX complex was evaluated by injecting the sample to HPLC.

$$\text{Drug loading efficiency}\;\left(\%\right)=\frac{\text{Weight of}\;\mathrm{DOX}\;\text{loaded}}{\text{Weight of}\;\mathrm{DOX}\;\text{and BPNSs}}\;X\;100$$

$$\text{Encapsulation efficiency}\;\left(\%\right)=\frac{\text{Weight of}\;\mathrm{DOX}\;\text{loaded}}{\text{Total Weight of}\;\mathrm{DOX}\;\text{added}}\;X\;100$$

#### 2.4.2 Polydopamine (PDA) coating on BP-DOX

The prepared BPDOX (2 mg) was added to 2 mL water. The BP-DOX solution was adjusted to alkaline pH (8.5) by using 0.01 M NaOH. To the above solution, 10 μL of dopamine hydrochloride of 100 mg/mL in water was added and stirred for 150 mins. The resulting PDA coated BP-DOX particles (BP-DOX@PDA) were collected by centrifugation (10,000 rpm for 10 min).

#### 2.4.3 Conjugation of Transferrin (TF) onto BP-DOX@PDA

1 mg of BP-DOX@PDA was added with tris-EDTA buffer and stirred the solution for 10 mins. The above solution was then added with 2 mg of TF to BP-DOX@PDA and continued the stirring for 2.5 h. The formed BP-DOX@PDA-TF particles were collected and lyophilized for 48 h and collected for further characterization.

#### 2.4.4 Estimation of TF conjugation using Bradford assay

A 10 mL TF stock solution was prepared in Tris-EDTA buffer at pH 7.4, and working standard solutions were subsequently prepared in the concentration range of 0.05 to 1.5 mg/mL. To prepare each working solution, 100 μL was transferred into separate volumetric flasks and added with 3 mL of Bradford reagent. The mixtures were mixed thoroughly and incubated until protein-dye complex formation and measured its absorbance at 595 nm (Xu et al., 2017).

#### 2.4.5 Characterization of the prepared BP-based nanoformulation

The particle size and PDI of the BPNSs were measured with dynamic light scattering (DLS). FTIR spectra of BP-based nanoformulation were obtained using the AT-FTIR scanned in a range of 400 to 4000 cm^−1^ to evaluate variations in the chemical characteristics of the produced BPNSs. XRD was employed to evaluate the crystalline characteristics of BP-based nanoformulation. Surface morphology of the nanoformulations were evaluated using TEM and SEM imaging.

### 2.5 Evaluation of the prepared BPNSs-based formulations

#### 2.5.1 In vitro photothermal effect

1 mL of the BP-based nanoformulation (BP-DOX@PDA-TF) aqueous solution was added into quartz vial, while water served as a control. The sample-containing cuvette was irradiated with NIR-II 808 nm laser (Laserglow technologies, North York, Canada). In order to evaluate the influence of concentration changes of BP-based nanoformulation, temperature fluctuations in solutions in a range between 100 and 1000 μg/mL were observed under 1.0 W/cm^2^ NIR irradiation. In addition, an BP-based nanoformulation (1000 μg/mL) was evaluated at different power intensities ranging from 250 to 2000 mV to assess variations in heat response. To get photothermal stability of the obtained BP-DOX@PDA-TF formulation (1000 μg/mL) was tested for on/off cycles by subjecting it to 808 NIR irradiation on and off for 10 min at 6 times. Furthermore, several formulations (BP, BP-DOX, BP-DOX@PDA, and BP-DOX@PDA-TF) were irradiated with 1.0 W/cm^2^ NIR irradiation for 10 min. Multi-Stem Digital Thermometer (Colorcon/Mextech) was employed for temperature measurement (Chen et al., 2021).

#### 2.5.2 Measurement of photothermal conversion efficiency (PCE)

The PCE (η) was measured by irradiating 200 μL BP-DOX@PDA-TF (200 μg/`mL) nanoparticles with 808 nm NIR laser (1 W/cm^2^) till the sample solution reached a phase of steady-state thermal condition. The PCE of BP-DOX@PDA-TF nanoparticles can be determined as follows according to previous reports:

$$\eta =\frac{\mathrm{hs}\left(T_{\max}-T_{\mathrm{surr}}\right)-Q_{\mathrm{Dis}}}{I\left(1-10^{-A_{808}}\right)}$$

where, h is the heat transfer co-efficient, s is the surface area of the container, T_max_-T_surr_ is temperature difference, T_max_ is maximum temperature, T_surr_ corresponds to environmental temperature, I is Laser power intensity (W), A_808_ denotes absorbance at 808 nm, and Q_Dis_ = heat dissipated because of light absorption by solvent and cuvette.

Thus, *h*s can be calculated as follows:

$$\mathrm{hs}=\frac{\mathrm{mC}}{\tau_{S}}$$

m = mass and C = water heating capacity. Where *τ*_*s*_ represents the slope of the linear relationship between cooling period time profile (Li et al., 2020).

#### 2.5.3 pH and NIR-activated DOX release profile

To analyze the DOX release pattern from BP-based nanoformulation (BP-DOX, BP-DOX@PDA, and BP-DOX@PDA-TF), 1 mg of DOX-loaded NPs were suspended in 1 mL phosphate-buffered saline (PBS) at pH 5.0 and 7.4, with a surfactant, tween 80 at 0.1 % *w*/*v* concentration. A dialysis membrane of MWCO 12500 was with formulation dispersion was immersed into 15 mL of buffers in an incubator at 37 °C. 500 μL of the receptor compartment buffer was removed and replaced it with PBS, at different time periods to measure the amount of released DOX using a designed analytical HPLC method at 234 nm. NIR 808 laser-induced DOX release was studied with 6 min laser irradiation at an intensity of 1.0 W cm^−2^ under similar conditions (Huang et al., 2021).

#### 2.5.4 Hemocompatibility study

Blood was removed from BALB/c mice by retro orbital method to a tube containing EDTA solution. The plasma from the blood sample was removed by centrifuging it at 4000 rpm and subsequent washing with PBS. The RBC concentrate was then added with 10 times volume of PBS 7.4. The diluted RBC was diluted 5 times with the formulation sample concentration ranging from 0.01 to 2.0 mg/mL. Positive control was made by adding 200 μL RBC suspension to 0.8 mL of 2 % *v*/v triton X100 and PBS 7.4 as negative control. The prepared samples, were kept in an incubator for 2 h and centrifuged at 3000 rpm for 5 mins to separate the RBC (Wu et al., 2021). % haemolysis was measured with UV spectrophotometer using the equation:

$$\%\text{haemolysis}=\frac{\text{Absorbance}\;\left(\text{Sample}\right)-\text{Absorbance}\left(-\mathrm{ve}\;\text{control}\right)}{\text{Absorbance}\;\left(+\mathrm{ve}\;\text{control}\right)-\text{Absorbance}\;\left(-\mathrm{ve}\;\text{control}\right)}\times 100$$

Structural changes of the formulation treated RBCs was checked with motic microscope.

#### 2.5.5 Chorioallantoic membrane (CAM) assay

The CAM assay is a method used to assess the effects of chemical agents/formulations on the developing, highly vascularized CAM. Fertilized chicken eggs were obtained, disinfected with 70 % ethanol, and incubated in a humidified environment. The eggs were kept horizontal and regularly rotated. On the eighth day a 1 × 1 cm^2^ window was cut into the eggshell, and DOX, BP, and BP-DOX@PDA-TF (1 mg/mL) BP-based nanoformulation (200 μL) prepared in 0.9 % saline solution was added to the CAM. Lenalidomide was considered as positive and normal saline (NS) as negative control. Following application, the egg's cavity was resealed with scotch tape and returned to the incubator. Changes in the blood vessel morphology were examined at 8 and 24 h after incubation (Raychaudhuri et al., 2021).

### 2.6 In vitro cell line studies

#### 2.6.1 Cell culture assay

##### 2.6.1.1 Cytotoxicity

The 4 T1 cell line, a murine breast tumour cell line derived from BALB/cf3H mice, was obtained from Elabsciences, Inc. The cytotoxicity of BP-based nanoformulation were evaluated using MTT assay. After trypsinization, 4 T1 cells were seeded onto a 96-well plate at 5 × 10^3^ cells/well and kept in an incubator for 24 h. The 4 T1 cells were then incubated with 50 μL of fresh medium containing samples of concentrations ranging from 0.001 to 100 μg/mL. After 48 h, media was discarded and added with 50 μL of MTT reagent (2 mg/ml). The samples were then kept for 4 h of incubation and added with 50 μL DMSO to solubilize the formazan crystals. OD (optical density) was evaluated at 540 nm in a microplate reader (ELx800).

##### 2.6.1.2 In vitro PTT study

Based on the results of cytotoxicity in 4 T1 cells produced by the test compounds, five concentrations were selected (100, 10, 1, 0.1, 0.001) to evaluate the extent of cytotoxicity in the presence of NIR radiation of 808 nm. 4 T1 cell line was seeded and incubated for 24 h. After refreshing the medium, the wells were added with samples at 37 °C for 4 h, and irradiated with 808 nm laser (1.0 W/cm^2^) for 10 mins incubated till 48 h (Phan et al., 2017; Xu et al., 2021).

Absorbance data were observed, and percentage tumour cell growth inhibition was measured using the following formula:

$$\text{Percentage growth inhibition}=\frac{\text{Absorbance of Blank}-\text{Absorbance of test}\;}{\text{Absorbance of Blank}}\;X\;100$$

#### 2.6.2 Cellular uptake evaluation

To understand the cellular uptake of nanoformulations, flow cytometry was employed. 4 T1 cells were seeded into 6-well plates and allowed to adhere for 24 h prior to treatment. Later, the free DOX, and BP-based nanoformulation were added (∼ concentration of 5 μg/mL). Followed by the removal of culture medium after 2 h of treatment, test samples were added, and the laser groups were exposed to NIR-II 808 nm laser for 5 min and kept in incubator for 2 h. The plates were taken and added with trypsin for the detachment of 4 T1 cells. It was then collected, centrifuged to separate free DOX and BP-based nanoformulation, and analyzed using a flow cytometer (BD Accuri). Untreated 4 T1 cells were used as a control group. DOX fluorescence intensity was measured with excitation and emission wavelengths of 488 and 593 nm, respectively (Peng et al., 2021).

#### 2.6.3 Evaluation of transferrin-mediated endocytosis

4 T1 cells were pre-incubated with a TF concentration of 2 mg/mL to estimate the targeting efficiency and specificity of the prepared nanoformulation’ s internalization through TF receptor-specific endocytosis. The 4 T1 cell was added with 1 ml free TF for 1 h and incubated with BP-DOX@PDA-TF formulation for 48 h. Followed by the incubation, the culture medium was removed and washed with PBS. The wells were then added with trypsin-EDTA (200 μL) harvested into a 12 × 75 mm polystyrene tube and separated by centrifugation. The fluorescence intensity of the collected 4 T1 cells were analyzed using flow cytometry (Bhatt et al., 2019).

#### 2.6.4 Apoptosis study

A dual staining strategy of utilizing acridine orange (AO) and ethidium bromide (EB) was employed for the morphological characterization of cellular apoptosis. IC50 concentrations of the nanoformulations were selected for the treatment. 1000 μL of EB (50 μg/mL) and 20 μL of AO (20 μg/mL) were used to stain 4 T1 cells for 10 mins. The morphological characteristics were focused using a fluorescence microscope after aspirating the stain solution. The fluorescence was observed via a filter with: (i) excitation and wavelength of 470/40 nm and 525/50 nm for AO and (ii) excitation wavelength 560/40 nm and emission wavelength 645/75 nm for EB.

#### 2.6.5 Cell cycle analysis of the black phosphorous-based formulations

Flow cytometry was used to assess the changes in the cell cycle characteristics caused by BP-based nanoformulation. The 4 T1 cells were incubated with plain DOX, BP-DOX and BP-DOX@PDA-TF. The supernatant was collected by centrifuging the incubated 4 T1 cells. The 4 T1 pellet was then fixed it using ethanol (70 %). The 4 T1 cells were harvested, centrifuged, and washed twice with cold PBS. The cell pellet was then resuspended in a PI/RNase staining solution and mixed thoroughly. The stained cells were incubated in the dark at room temperature for 15 min. Finally, the samples were analyzed by flow cytometry to assess cell cycle distribution (Sahrayi et al., 2022).

### 2.7 In vivo preclinical studies

Female BALB/c mice (6–8 weeks old, 18–25 g) were obtained from a certified animal supplier and used for the study. Prior to the start of the experiment, all animals were housed under standard laboratory conditions (12-h light/dark cycle, temperature 22 ± 2 °C, relative humidity 50–60 %) with ad libitum access to food and water. A two-week acclimatization period was provided to allow the animals to adjust to the laboratory environment. The experimental protocol was reviewed and approved by the Institutional Animal Ethics Committee (IAEC), Manipal Academy of Higher Education, Manipal, India (Reg No: 94/PO/ReBi/S/99/CPCSEA), and all procedures were conducted in accordance with CPCSEA guidelines. Mice were randomly assigned to experimental groups (*n* = 3 per group), and all efforts were made to minimize suffering. The allocation and data collection were conducted in a blinded manner wherever applicable. No animals were excluded from the analysis unless stated otherwise.

### 2.8 In vivo pharmacokinetics

To measure the pharmacokinetics parameters, free DOX (Dose: 5 mg/kg), BP-DOX, and BP-DOX@PDA-TF (at an equivalent dose of 5 mg/kg) were given by i.v. route via tail vein injection into the mice (*n* = 3). Blood sample was collected in EDTA solution containing tubes at intervals ranging from 5 min to 48 h post-administration and then centrifuged to separate the plasma (Zeng et al., 2019). 5 μL of internal standard, daunorubicin (DAN) was added to the plasma and precipitated by adding 200 μL of acetonitrile, mixed well, and separated the supernatant containing DOX by centrifugation. To evaluate the plasma drug concentrations, an RPHLC technique was employed to quantify DOX concentration at wavelength of 234 nm (Bobde et al., 2021).

### 2.9 Pharmacodynamic studies

Female BALB/c mice were given a subcutaneous injection of 4 T1 cells (1.5 × 10^6^ cells) in 100 μL of cold PBS, into the dorsal flank. The breast tumour appeared in 10–14 days while the mice were under close observation. Using a vernier caliper, the length and width were measured, and the tumour volume was measured. When the tumour volume reached about 75 to 100 mm^3^, the mice were grouped randomly to 10 groups (n = 3):

Group I: Saline (i v injection).

Group II: Saline + NIR (i v injection).

Group III: BPNSs (Dose: 5 mg/Kg) (i v injection).

Group IV: BPNSs + NIR (Dose: 5 mg/Kg) (i v injection).

Group V: DOX (Dose: 5 mg/Kg) (i v injection).

Group VI: DOX + NIR (Dose: 5 mg/Kg) (i v injection).

Group VII: BP-DOX (Dose: 5 mg/Kg eq. to DOX) (i v injection).

Group VIII: BP-DOX + NIR (Dose: 5 mg/Kg eq. to DOX) (i v injection).

Group IX: BP-DOX@PDA-TF (Dose: 5 mg/Kg eq. to DOX) (i v injection).

Group X: BP-DOX@PDA-TF + NIR (Dose: 5 mg/Kg eq. to DOX) (i v injection).

The mice groups were administered with 100 μL of NS, BP, DOX, BP-DOX and BP- DOX@PDA-TF by i.v. injection via tail vein at an dose 5 mg/kg equivalent to DOX [30]. After 2 h of injection, the laser irradiation groups were exposed to 808 nm NIR laser (1 W/cm^2^) for 5 min. The starting day of the treatment was considered as 0th day and checked for body weight in every day and tumour volume in every 2 days. The following equation was used for the measurement of tumour volume (V):

$$\text{Volume}\;\left({\mathrm{mm}}^{3}\right)=\frac{{\text{Width}}^{2}X\;\text{Length}}{2}$$

On the last day of the treatment (Day 14), the BALB/c mice were euthanized by cervical dislocation and major organs were collected, washed and fixed with 4 % paraformaldehyde for Hematoxylin and Eosin (H&E) staining.

#### 2.9.1 In vivo IR thermal images

In vivo thermal imaging of the BP nanoformulation-treated animals was captured by irradiating 4 T1-tumour bearing mice. The mice were administered via i.v. route with saline, DOX, BP, BP-DOX and BP-DOX@PDA-TF of 100 μL volume. Before drug administration, the mice were given a ketamine-xylazine mixture anesthesia to make them immobile and comfortable. Thermal imaging camera (FLIR ONE Gen 3) sensitive to NIR wavelengths were positioned to capture real-time thermal data from the mice during laser irradiation. The NIR laser (Laserglow technologies, North York, Canada) was then used to the target tissue area, with continuous monitoring of temperature changes to precisely control laser parameters and minimize damage to surrounding tissues. Thermal images and temperature data were analyzed post-procedure to assess tissue response and treatment efficacy. Our findings contribute to the understanding of tissue responses to thermal stimuli and the development of novel therapeutic interventions (Jia et al., 2020).

#### 2.9.2 Histopathological analysis in tumour tissues and other organs

Three BALB/c mice from each group were sacrificed after 14 days of treatment. The tissues (liver, spleen, lung, heart, kidney, and breast tumour) were isolated and fixed with 10 % formalin for 24 to 48 h. The tissue slides were examined using the LX-500 LED trinocular Research microscope, and pictures were captured using the MiaCam CMOS AR 6pro microscope camera coupled to image AR pro software. The slides were graded according to tumour cells, necrosed areas, malignant features like nuclear pleomorphism and hyperchromatism, inflammation, muscle invasion and mitotic count counted in 10 consecutive high-power fields (HPFs).

#### 2.9.3 Variation in body weight

During the study period of BP-based nanoformulation, the body weight of the animals was checked at specified time durations.

#### 2.9.4 Blood parameters estimation

A total of about 100 μL of blood was collected from the retroorbital route and collected into a tube with 10 μL of EDTA for a complete blood count such as RBC, WBC, platelets and hemoglobin analysis using Nihon Kohden blood analyzer (celltac alpha-MEK-6550, India).

#### 2.9.5 Biochemical parameters estimation

The impact of the formulation on key organs, including the liver (assessed by measuring alanine aminotransferase [ALT], albumin, total bilirubin, alkaline phosphatase [ALP], aspartate aminotransferase [AST], and total protein levels in the blood) and the kidneys (evaluated by measuring creatinine and urea in the blood), were analyzed using a semi-automated analyzer (STAR analyzer).

#### 2.9.6 IL-6 estimation by ELISA analysis

The IL-6 levels in the samples were determined using the Invitrogen IL-6 ELISA kit (Mouse IL-6 ELISA Kit, High Sensitivity) according to the instructions. Before using any of the reagents or samples, they were all brought to room temperature. Standards were reconstituted and serially diluted to create a standard curve. Incubate 100 μL of standards, controls, and samples on a pre-coated 96-well plate for 2 h by shaking gently. The wells were then aspirated and with 0.3 mL of wash buffer (4 times). After incubating with biotin-conjugated detection antibody (100 μL) to each well, the plate was gently shaken and kept for an hour at room temperature (RT). The wells were washed and added with 100 μL of streptavidin-HRP and kept for 45 mins in shaking incubator. After washing the wells, 100 μL of TMB substrate solution was applied to each well and incubated for 15–30 min. The wells were then added with a stop solution to halt the reaction and observed absorbance at 450 nm. The IL-6 level was measured by extrapolating the values of IL-6 standard curve.

#### 2.9.7 TNF-α estimation by ELISA analysis

To estimate TNF-alpha with the Invitrogen ELISA kit (Mouse TNF alpha ELISA Kit, High Sensitivity), plasma samples were collected. All reagents, including washing buffer, standard dilutions, and detection antibodies, were prepared according to the kit instructions. Standards, samples, and controls were placed in duplicate wells with 100 μL in each and incubated at RT for 2 h to allow TNF-alpha to bind to immobilized antibodies. The wells were washed 4 times with wash buffer to eliminate any unbound compounds. Next, 100 μL of biotinylated anti-TNF-alpha antibody was added to each well, and incubated for another hour at RT. To eliminate any remaining unattached detection antibody, the wells were washed again. Then, Streptavidin-HRP conjugate (100 μL) was incubated and kept at RT for 0.5 h, followed by further washes to remove any unbound conjugate. To develop colour, 0.1 mL of TMB substrate was added and 100 μL of stop solution was added to stop the reaction. The colour of the solution was changed from blue to yellow. Finally, absorbance at 450 nm was analyzed with background absorbance removed at 570 nm or 630 nm as needed. The data were analyzed by creating a standard curve using the standards' absorbance values, and the absorbance values were compared to the standard curve to calculate the TNF-alpha concentration. This complete approach used the Invitrogen ELISA kit to accurately estimate TNF-alpha levels in a variety of biological samples.

#### 2.9.8 Ki-67 estimation

The Ki-67 levels of the collected tissues were measured according to the established protocols. In summary, tumour tissue sections were subjected to treatment with block buffer for 1 h. Afterwards, the samples were refrigerated at 4 °C overnight while being treated with antibody targeting Ki-67. The incubated tissue slices were then subjected to three rounds of PBS washing. Subsequently, Alexa Fluor Plus 488, a secondary antibody was introduced and incubated for 2 h at 25 °C in dark. Ultimately, the tissue slices were washed and observed under a fluorescence microscope (Itoo et al., 2025).

#### 2.9.9 ROS detection assay

The production of ROS in breast tumour region was measured with a fluorescent probe DCFH-DA. BALB/c mice with 4 T1 tumour xenografts received an intratumoural injection of 50 μL of DCFH-DA (concentration: 25 μM) after 14 days after administration. After 30 min, isoflurane inhalation was employed for mice anesthesia and in vivo imaging system (IVIS® Lumina III) was used for the live imaging of ROS production. The animals were sacrificed, excised the tumours, sectioned into 5 μm thickness slices with a cryostat (Leica biosystems, Germany), and observed under a fluorescence microscope (Bobde et al., 2021).

#### 2.9.10 TUNEL assay

A TUNEL test was employed to evaluate the amount of apoptosis in TNBC tumour tissue. Excised tumours were sectioned into 5 μm thickness slices with a cryostat followed by treatment with tunel reagent and storage in 4 % paraformaldehyde. The degree of apoptosis in the tumour slice was measured in accordance with manufacturer guidelines. Blue and green filters were utilized in the fluorescence microscope to observe the stained tumour segments. ImageJ software was used for the processing and analysis of the image (Bhatt et al., 2020).

### 2.10 Biodistribution study

In vivo biodistribution study of plain DOX, and BP-DOX@PDA-TF were monitored with an in vivo imaging system. In brief, plain DOX, and BP-DOX@PDA-TF were injected into female BALB/c nude mice xenograft model bearing 4 T1 tumours via i.v. route. Using IVIS® containing 620 nm and 780 nm filters respectively for excitation and emission, tumour accumulation trends were examined across various time periods following injection. In tumour mice model, the biodistribution pattern of the samples were observed for 36 h. The fluorescent signal of DOX was taken after 0.5, 1, 3, 6, 12, 24, and 36 h after injection (Hao et al., 2015).

## 3 Results and discussion

### 3.1 Preformulation studies of drug and bulk BP

#### 3.1.1 Identification of drug by DSC

**Fig. 1:**
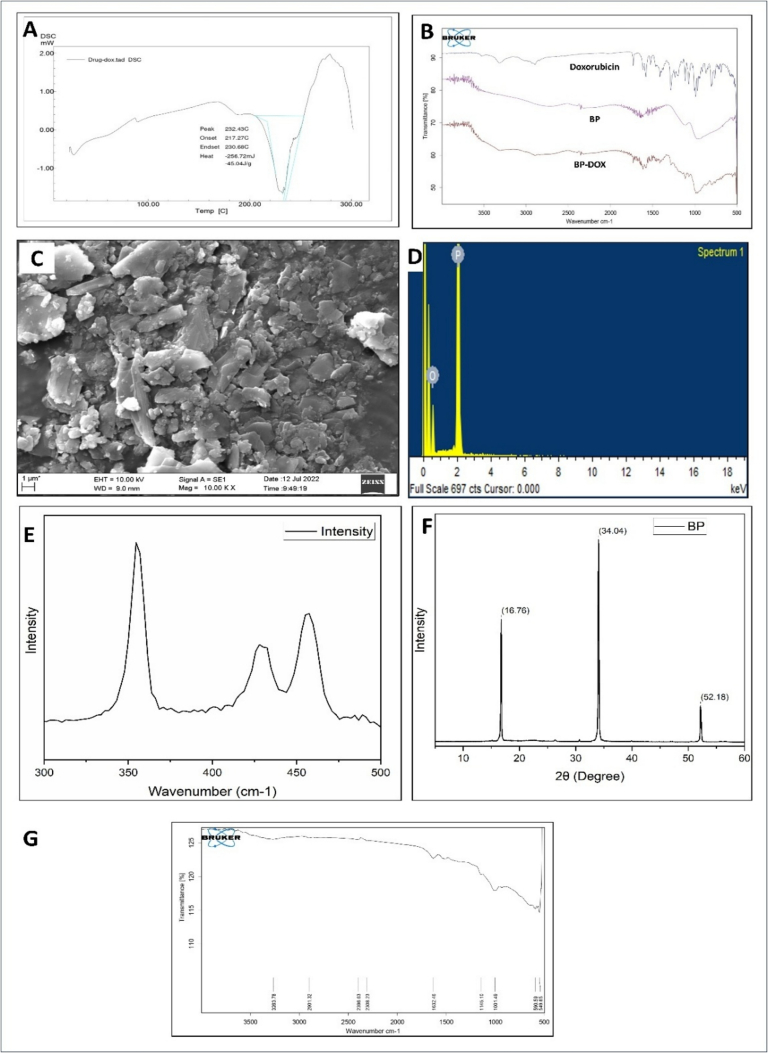
Characterization of DOX and bulk BP. (A) DSC thermogram, and (B) FTIR spectrum of Doxorubicin, (C)FTIR spectrum, (D) EDS spectrum, (E) SEM image, (F) XRD pattern, (F) Raman spectrum, and (G) FTIR spectrum comparison of bulk black phosphorous.

A DSC analysis of pure DOX was performed to examine its thermal characteristics and identity. The DSC thermogram revealed a broad endothermic peak ranging from 210 to 240 °C, which corresponds to DOX's melting point (Gao et al., 2019). This peak is critical for understanding the drug's thermal behaviour because it represents the temperature at which DOX transitions from a solid to a liquid state (Fig. 1.A.). The thermal profile obtained from the DSC analysis provides valuable insights into DOX's stability under varying temperature conditions, which is critical for handling and storage.

#### 3.1.2 Identification of drug by FTIR

The FTIR spectrum of DOX reveals several characteristic peaks that correspond to specific functional groups, providing insights into its molecular structure (Fig. 1.B.). The peak at 3315.29 cm^−1^ suggesting the N—H stretching vibration, which is typical for amines and is essential for confirming the amino group in DOX. The O—H stretching peak at 3526.62 cm^−1^ suggesting hydroxyl groups, which are critical for the drug's solubility and interaction with biological targets. The peaks at 2896.09 cm^−1^ and 2843.80 cm^−1^ correspond to C—H stretching, reflecting the aliphatic and aromatic hydrogen atoms in the DOX structure. The C=O stretching peak at 1729.10 cm^−1^ is significant as it indicates the presence of carbonyl groups, which are vital for the drug's reactivity and interaction with cellular components. Additionally, the peaks at 1613.18 cm^−1^, 1581.08 cm^−1^, and 1408.06 cm^−1^ are associated with C=C ring stretching, characteristic of aromatic systems, confirming the presence of the anthraquinone structure in DOX. The C-O-C stretching peaks at 1144.40 cm^−1^ and 1069.91 cm^−1^ further support the identification of ether or ester functionalities in the molecule. Finally, the peaks at 799.40 cm^−1^ and 685.91 cm^−1^ correspond to C=H bending and C=C ring bending, respectively, which are also indicative of the aromatic nature of DOX. Overall, the FTIR analysis provides a comprehensive characterization of DOX, confirming its structural integrity and functional groups critical for its pharmacological activity (Das et al., 2010).

#### 3.1.3 Characterization of bulk BP

(i) Microscopy characterization

Fig. 1.C. displays the obtained SEM image of the BP. The layer architecture of BP is readily observed in the SEM images. SEM analysis provided high-resolution imaging of the surface topography of bulk BP samples.

(ii) Spectroscopy characterization

By using a semi-quantitative EDS analysis, the chemical composition of the bulk BP was found to be phosphorous (76.92 wt%) and oxygen (23.08 wt%) as illustrated in the Fig. 1.D. The presence of oxygen was identified as a result of BP being handled and characterized in the presence of air. We used Raman spectrophotometer to evaluate the crystalline orientation of the obtained BP. Fig. 1.E. shows the Raman spectra for the bulk BP. Three major peaks were observed in this spectrum at 354.63 cm^−1^, 428.01 cm^−1^, and 457.53 cm^−1^ represents the A^1^_g_, B_2g_, and A^2^_g_ phonon modes of bulk black phosphorous. This result confirms the orthorhombic crystalline structure of bulk BP. Within the same plane of the layers, atomic oscillations indicating B^2^g and A^2^g vibrational modes. In the A^2^g mode, phosphorus atoms vibrate in a zigzag pattern, while in the B^2^g mode, they oscillate in the armchair direction. Additionally, atomic oscillations related to the A^1^g vibrational mode occur out of the plane.

We used Rigaku Miniflex 600 (5th gen) X-ray diffractometer to check the crystalline nature of bulk BP. Fig. 1.F. represents the XRD spectrum, where intense peaks were observed at 16.76^°^, 34.04° and 52.18^°^, which are corresponding to the reference BP. In FTIR spectrum Fig. 1.G., at wave number positions of 1001.49 and 590.59 cm^−1^, the two weak characteristic bands of bulk BP are detected, which is an indication P—O stretching and the P=O bending peaks, respectively.

#### 3.1.4 Drug-excipient compatibility study

At wave number positions of 1001.49 and 590.59 cm^−1^, the two weak characteristic bands of bulk BP are detected, which can be ascribed respectively to the P—O stretching P=O bending vibrations, respectively. The physical mixture of bulk BP with DOX spectra showed that the distinctive peak of DOX remained unchanged, which is an indication of no compatibility concerns Fig. 1.B.

### 3.2 Synthesis of BPNSs from bulk BP and its characterization

**Fig. 2:**
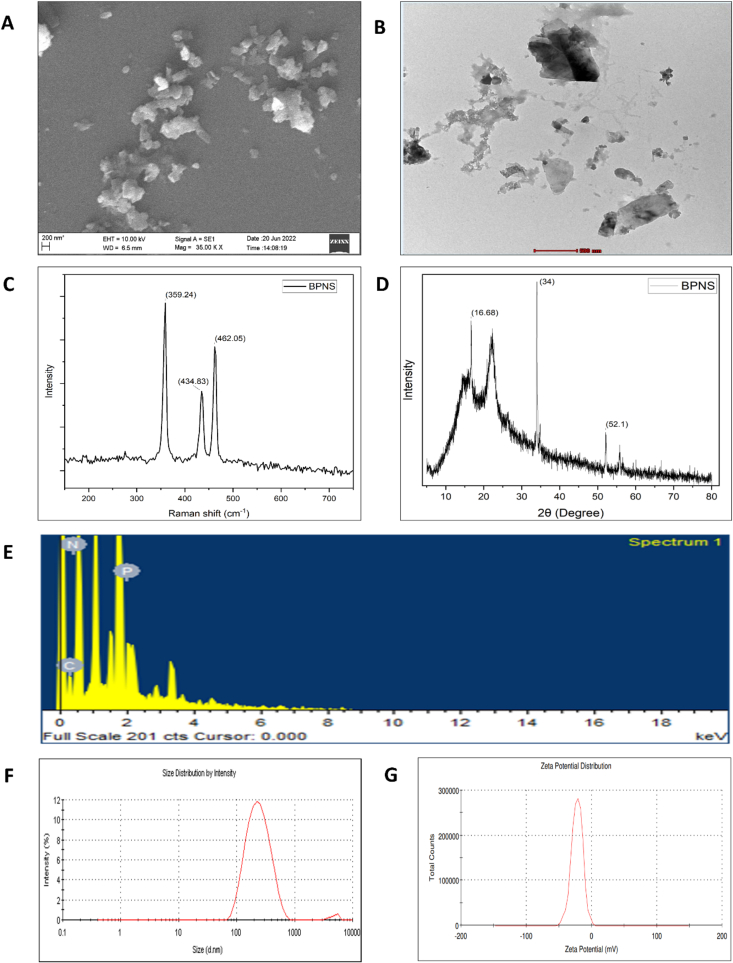
Characterization of the exfoliated BPNSs (A) SEM Analysis, (B) TEM analysis (Scale bar = 500 nm), (C) XRD pattern, (D) Raman spectrum, (E) EDS, (F) Particle size, and (G) zeta potential.

The BPNSs were synthesized via liquid exfoliation technique from the obtained bulk BP and characterized using different methods. The SEM micrographs obtained clearly shows layer architecture of BP. The SEM images clearly confirm the appropriate BP exfoliation to nanosheets with a particle size of 226.5 nm. The obtained SEM image is given in the Fig. 2.A. TEM analysis was used to study the morphological properties and structure of the exfoliated BPNSs. TEM image shown in the Fig. 2.B. suggests that BPNSs exhibit sheet-like structure with particle diameter of around 200–250 nm, being consistent with results from SEM analysis.

The XRD diffraction of exfoliated BPNSs is given in Fig. 2.C. In case of bulk BP, the d-spacing of (20) peak present at 2θ of 16.75° was found to be 5.28 A^°^. Followed by exfoliation of bulk BP 2θ of the peak (20) was shifted to 16.66^°^, which corresponds to the d-spacing of 5.31 A^°^. This change in d-spacing value shows that the NMP molecules efficiently interacted with the bulk BP layers during ultrasonic exfoliation. The XRD results suggested that NMP is an ideal solvent of choice for weakening the Van der Waals.

Fig. 2.D. shows that the raman spectra of the BPNSs is different from that of bulk BP. Three distinguishable Raman peaks were observed in the spectral range of 300 to 500 cm^−1^. BPNSs exhibited well-defined peaks at 359.24 cm^−1^, 434.83 cm^−1^, and 462.05 cm^−1^ indicates the A^1^_g_, B_2g_, and A^2^_g_ phonon modes respectively and this is in good agreement with the literatures. The phosphorous, carbon and nitrogen are found in the obtained EDS spectrum (Fig. 2.E.) of exfoliated BPNSs. The result suggesting that the nanosheet is BP without significant oxidation, observed through the NMP-ice-mediated exfoliation technique. The vibrational modes blue-shift indicates thinner BP layers in comparison with to bulk BP. The BPNSs showed a particle diameter of 213 nm and zeta potential of −22.5 mV. The PDI of nanosheets has been 0.213. The result suggested that the nanosheets are efficiently stable against further aggregation in the solution. The P—O bond on the surface of nanosheets is what causes the zeta potential to have a negative sign. It is vital to notice that the size and charge of the nanosheets have a significant impact on how proteins and cells interact with them. According to reports, negatively charged nanoparticles are more suited for therapeutic applications because they have longer blood circulation times and avoid interactions with similarly charged serum proteins as given in the Fig. 2.F. and G.

### 3.3 Formulation and characterization of BP-based nanoformulation

**Fig. 3:**
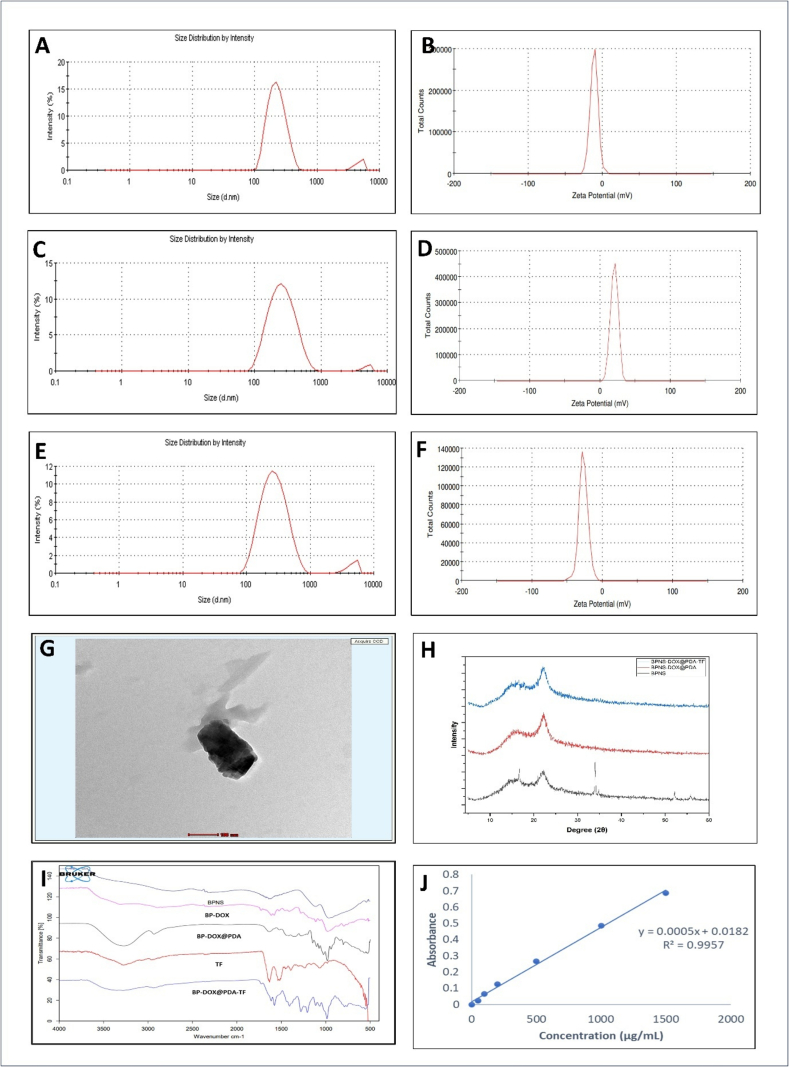
Characterization of BP-based nanoformulation. DLS size measurement of (A) BP-DOX, (C) BP-DOX@PDA, and (D) BP-DOX@PDA-TF, Zeta potential of (B) BP-DOX, (D) BP-DOX@PDA, and (F) BP-DOX@PDA-TF, (G) TEM image of BP-DOX@PDA-TF (Scale bar = 100 nm), (H) XRD spectrum, (I) FTIR spectrum, and (J) Standard plot of TF using Bradford assay.

It was found that the prepared formulation exhibited an encapsulation efficiency of 91.56 % *w*/w and a percentage loading efficiency of 61.04 % w/w. When BP-DOX was measured using DLS, its size increased to 234 nm (Fig. 3.A) as compared to plain BPNSs (213 nm). Moreover, the zeta potential (ZP) measurement demonstrated a significant difference of −11.2 mV when compared to bare BPNSs (−22.5 mV) (Fig. 3.B). The observed significant increase in ZP values between DOX-loaded and blank nanosheets may be attributed to DOX adsorption on the surface of the BPNSs (Zhao et al., 2015). The ZP was shifted it to +20.1 mV with a hydrodynamic size of 251 nm followed by PDA coating. This change in the surface charge can be attributed due to the hydroxyl group deprotonation of PDA layer (Nie et al., 2017). No particle aggregation had taken place and aqueous dispersibility had been preserved (Fig. 3.C and D). The altered surface characteristics of the particles after coating were further supported by a difference in net surface charge (ζ-potential) (Pada et al., 2019). The DLS size measurement (Fig. 3.E) of the prepared BP-DOX@PDA-TF was found to be 260.9 nm, which confirms the successful conjugation of TF. TF has been utilized for the surface functionalization of BP-DOX@PDA through electrostatic interaction between BP-DOX@PDA (positively charged) and TF (negatively charged). The ZP of the BP-DOX@PDA-TF (Fig. 3.F) was found to be −27.1 mV, which was more negative than that of BP-DOX@PDA (20.1 mV). Modifications to TF or the removal of the amine group may have an impact on the net charge of the nanoparticles, which may result in the negative charge transferrin conjugation to the BP-DOX@PDA surface (Jang and Oh, 2017).

The morphology of the prepared BP-based nanoformulation was evaluated via TEM (Fig. 3.G) revealed a sheet like structure with a particle size of less than 200 nm. The slight difference in particle size observed between TEM and DLS measurements is expected to be due to the effects of the dispersant on the hydrodynamic diameter of the BPNSs. In comparison with plain BP, the BP-DOX@PDA-TF demonstrated a layer thickness of about 8.6 ± 4 nm at the particle surface. The layer structure on the nanosheet surface is an indication of efficient functionalization of nanoparticle with PDA and conjugation with TF.

PDA is an excellent biopolymer used for the surface functionalization of various nanoplatforms, which was invented based on the mechanism of mussel adhesion. The characteristic features of PDA such as greater adhesiveness, efficient bioavailability, facile synthesis requirements and excellent photothermal conversion efficiency makes PDA-modified nanoplatforms as an ideal choice to use as drug carriers. Dopamine undergoes oxidative self-polymerization into PDA under alkaline condition, eventually attaching to the surface of NPs. Due to its reactivity with thiol and amine groups, PDA coating improved physiological stability, likely acting as a strong connection between NS and protein-based ligand. DLS and zeta potential were checked after each functionalization process to see whether it was successful and to make sure that The XRD patterns (Fig. 3.H) of BP-DOX@PDA show that the phase of BP is preserved, and there is some widening of the diffraction peaks, indicating a reduction in crystallite size (Ramireddy et al., 2016).

DOX's FTIR spectra (Fig. 3.I) revealed a variety of distinctive peaks, including 2900 cm^−1^ (C—H stretching), 866 cm^−1^ and 802 cm^−1^ (N—H wagging), 1228 and 1408 cm^−1^ (Alcoholic O—H and C—C stretching respectively), and 799 cm^−1^ (N—H and NH_2_ wagging). When BP-DOX is compared to bare DOX, the C—C stretching peak at 1070 cm^−1^ is similar in both spectra. When doxorubicin and BP-DOX were compared, it was found that the peaks at 866 and 803 cm^−1^ were present in DOX loaded BPNSs but were somewhat decreased. In DOX loaded BPNSs, the DOX peak at 1228 cm^−1^ was seen, indicating the loading of doxorubicin. A change from 802 to 799 cm^−1^ was also observed, suggesting a bond between DOX and BPNSs (Kayal and Ramanujan, 2010). The FTIR spectrum confirmed the effective PDA coating to the nanoparticle. The intense and broad peak appeared in between 3150 and 3600 cm^−1^ demonstrating the stretching vibrations of N-H/O-H. Another additional peak at around 1500 cm^−1^ demonstrating -NH bending vibrations as benzene ring in PDA (Shen et al., 2015). Transferrin showed bands at approximately 1634, 1527, and 1236 cm^−1^ indicating amides I, II, and III, respectively. As a result of transferrin being effectively conjugated to the surface of BP-DOX@PDA, the band intensity was reduced (Heggannavar et al., 2019).

Bradford assay was used to estimate the extent of TF conjugation onto PDA coated BP-DOX. In different fields of biology and biochemistry, accurate and rapid estimation of protein concentration is essential. Bradford assay is a standard technique for measuring protein levels. In contrast, the Lowry method is more complex, slower, and less sensitive. The Bradford assay depends on the interaction of Coomassie Blue G250 dye with proteins (Chial et al., 1993). A calibration curve of TF was created by graphing concentration of TF against absorbance using a UV spectrophotometer. The standard curve demonstrated a strong linear relationship with an R^2^ near to unity (Fig. 3.J). The TF conjugation was performed at various stirring time such as 30 mins and 150 mins. Conjugation efficiency of TF onto the PDA coated BP-DOX was 3 % and 26 % at 30 mins and 150 mins respectively.

### 3.4 Evaluation of the prepared BPNSs-based formulations

#### 3.4.1 In vitro photothermal effect

**Fig. 4:**
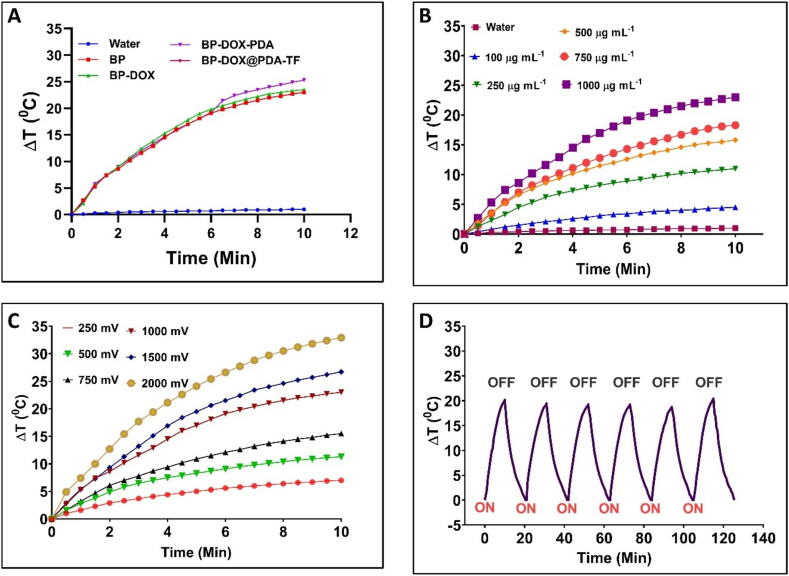
Photothermal curves of BP-based nanoformulation explaining its Photothermal characteristics. (a) Different formulations, (b) BP nanoformulations of concentrations ranging from 0.1 to 1.0 mg/mL, (c) Under different NIR power intensities ranging from 250 to 2000 mV, and (d) Exposure of BPNSs suspension towards six continuous on/off NIR laser irradiation cycles at laser intensity of 1.0 W/cm^2^, confirming good photothermal durability.

The BP-based nanoformulation were evaluated for its photothermal efficiency by irradiating them with near-infrared radiation of 808 nm wavelength. The as-synthesized BP-based nanoformulation was checked for its concentration-mediated temperature changes towards NIR irradiation (Fig. 4). The BPNSs formulation of different concentrations of 0, 0.1, 0.25, 0.50, 0.75, and 1 mg/mL were dispersed in water and irradiated with near-infrared light of 1.0 W/cm^2^ laser intensity (NIR-II, 808 nm). The difference in the heat response was measured in terms of NIR exposure duration. At 0.1 mg/mL, BP-DOX@PDA-TF showed a temperature rise of 4.5 °C. Whereas BP-DOX@PDA-TF demonstrated a significant increase in the temperature of 23 °C at a high concentration (1.0 mg/mL). It shows the effectiveness of BP-DOX@PDA-TF for effective transformation of NIR laser light into temperature.

The effect of NIR intensity on heat conversion property of the prepared BP-based nanoformulation was evaluated by irradiating 1 mg/mL of BP-DOX@PDA-TF with various NIR power in intensities ranging from 0.25 to 2 W/cm^2^. The lowest intensity of 250 mV exhibited a reduced temperature difference of 7 °C in comparison with 20 W/cm^2^ power intensity which exhibited an increased temperature difference of 32.9 °C. This notable difference in the temperature shows NIR-II intensity-mediated photothermal activity of BP-DOX@PDA-TF. Another important parameter for determining the photothermal performance of a photo-sensitive material is its photothermal stability. The continuous six on/off NIR-II irradiation resulted negligible temperature fluctuations, proving that the BP-DOX@PDA-TF demonstrated excellent photothermal stability.

#### 3.4.2 Estimation of photothermal conversion efficiency (PCE)

The PCE of the BP-based nanoformulation was assessed to evaluate the ability of the photothermal agent to convert light into heat energy. The changes in heat rise of BP-based nanoformulation (BP, BP-DOX, and BP-DOX@PDA-TF) in an aqueous dispersion (concentration of 1000 μg/mL) was measured. Laser exposure caused BP-DOX@PDA-TF quickly to reach 48.4 °C and down to room temperature without laser radiation. DOX-loaded BPNSs's PCE was determined to be 10.4 %. Photothermal efficiency of 11.27 % is achieved by BP-DOX coated with PDA and TF. The higher PCE value of BP-DOX@PDA-TF demonstrated that the PDA modification was advantageous in enhancing the photothermal features.

#### 3.4.3 pH and photothermal-mediated DOX release pattern

**Fig. 5:**
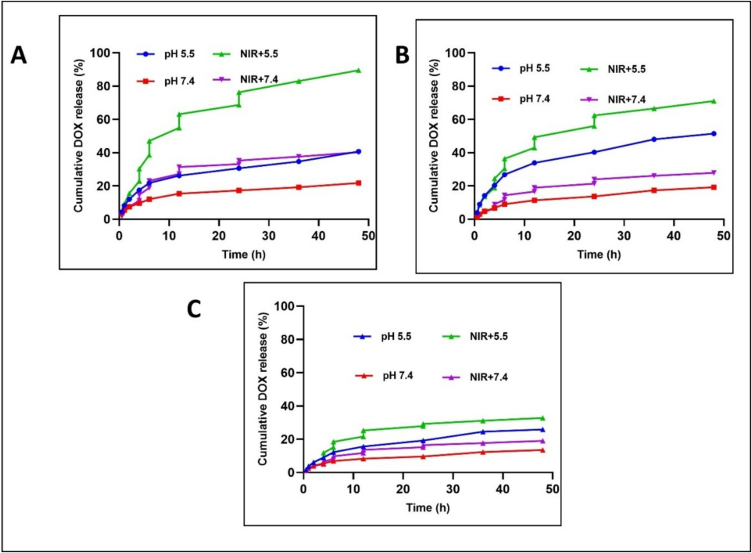
In vitro DOX release profile of (A) BP-DOX, (B) BP-DOX@PDA, and (C) BP-DOX@PDA-TF at different pH (7.4 and 5.5) with and without NIR irradiation.

The sustained and controlled DOX release characteristics of BP nanoformulation was carried out at pH 7.4 and 5.0, to provide a normal physiological condition and tumour lysosomal/endosomal condition respectively (Fig. 5). Over 48 h at pH 7.4, BP-DOX, BP-DOX@PDA, and BP-DOX@PDA-TF demonstrated about 24.39 %, 19.17 %, and 13.52 % of DOX release respectively. When exposed to tumour microenvironment of pH 5.0, DOX release was substantially enhanced to approximately 40.73 %, 51.47 %, and 27.91 %, showing pH-responsive drug release from BPNSs. DOX release was increased in an acidic environment, due to DOX protonation and collapse of PDA coating.

On irradiation with NIR-II radiation for 6 min, DOX release rose notably but decreased in non-irradiated groups. Following 5 cycles of NIR laser exposure, up to 32.78 % of the drug release was observed in BP-DOX@PDA-TF. The BP-induced local temperature rise under irradiation can be attributed to NIR-induced accelerated drug release. As a result, BP-DOX@PDA-TF proved capable of effective drug release in the low pH of tumour, predominantly when activated by NIR stimulation, enhancing the effectiveness of antitumour medications while minimizing their negative effects. This is consistent with the study by Gao et al., where poly(2-ethyl-2-oxazoline)-modified black phosphorus nanosheets were developed as pH-responsive and photothermal-triggered drug delivery platforms (Gao et al., 2019).

#### 3.4.4 Hemocompatibility study

**Fig. 6:**
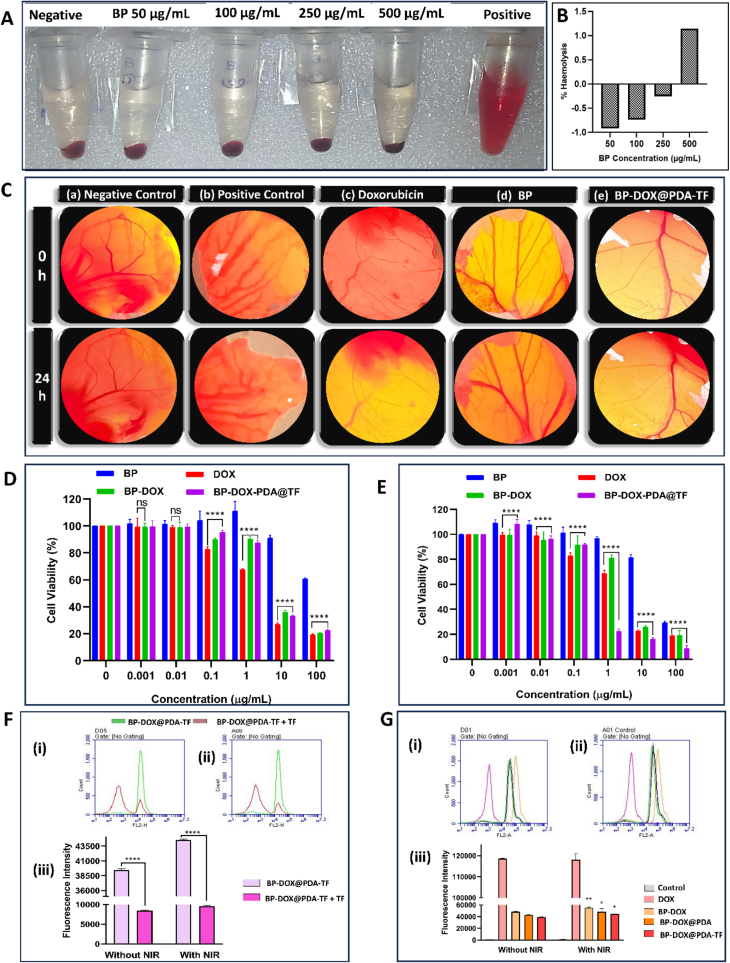
Toxicity evaluation and cellular uptake of BP-based nanoformulation. Hemolysis activity of exfoliated BPNSs. (A) Hemolysis observation after treatment with BPNSs (50–500 μg/mL concentration) in comparison with the control groups. (B) Percentage haemolysis observation of exfoliated BPNSs at various concentrations, indicating hemocompatibility. (C) CAM assay observation of BP-based nanoformulation in comparison with free DOX and control groups. The 4 T1 cell viability by MTT assay (D) Chemotherapy with no irradiation and (E) synergistic therapy with chemotherapy and NIR exposure (1 W/cm^2^ for 10 min). (F) Cellular uptake study histogram results of (i) NIR laser non-irradiated groups and (ii) irradiated groups; and (iii) Quantitative analysis of the fluorescence intensity in comparison with NIR-exposed with non-irradiated groups. (G) Evaluation of transferrin receptor-mediated endocytosis. Representative histogram plot of (i) NIR non-irradiated groups and (ii) NIR irradiated groups; and (iii) Fluorescence intensity comparison of NIR-irradiated groups with non-irradiated groups.

RBCs are the predominant cells in blood, constituting 99 % of it, and are crucial for oxygen transport. When nanomaterials are introduced intravenously, RBCs are among the first to interact with them. The current evaluation is performed to measure the influence of BPNSs on hemolysis. Purified RBCs were incubated with nanosheets at 37 °C (600 rpm) to enhance the RBC-nanosheet contact. Inadequate contact can lead to the hemoglobin outflow, signalling hemolysis. After treating RBCs with BPNSs, the upper liquid appeared a light yellowish, as illustrated in Fig. 6.A, with negligible hemoglobin outflow at a high concentration of 500 μg/mL concentration. An increase in the extent of hemolysis was observed in higher concentrations of BPNSs. However, no substantial hemolysis (<5 %) was detected (Fig. 6.B) when BPNSs were in contact with RBCs.

#### 3.4.5 Chorioallantoic membrane (CAM) assay

The CAM assay is a crucial method in pharmaceutical investigations for assessing formulation toxicity. CAM test is a cost-effective and ethical approach for the immediate evaluation of possible vascular injury and effects on fetal growth. By examining factors like vascular and embryotoxicity, this assay allows for early detection of possible formulation concerns. This assay allows a comparison of the toxicity perspectives of different drugs and pharmaceutical substances, assisting in the thorough evaluation of the safety aspects of the samples [51]. Plain BPNSs administered groups showed no substantial influence on CAM, with no signs of coagulation, hemorrhage and vascular morphology in comparison with the positive control lenalidomide as shown in Fig. 6.C. This indicates that the exfoliated BPNSs are safer and is biocompatible with no damage to the CAM. In addition, no embryotoxicity was observed during entire test period. The CAM assay demonstrated a significant different in the vascular structure of CAM treated with BP-DOX@PDA-TF with equivalent DOX concentration of 200 μg and free DOX. The BP-based nanoformulation administered CAM showed no signs of toxicity to the CAM and it was intact. However, the free DOX (200 μg) administered eggs showed a notable sign of toxicity such as coagulation, bleeding, and embryo toxicity. These observations suggest that free DOX demonstrates a number of negative effects, which can be minimized by loading it onto nanocarriers.

### 3.5 In vitro cell line studies

#### 3.5.1 MTT assay

The cytotoxicity of the prepared BP-based nanoformulation was evaluated in terms of chemo-photothermal activity by MTT assay. As illustrated in Fig. 6.D and E, after 48 h of incubation with 100 μg/mL of BPNSs, 4 T1 cells were viable at around 60 %, showing negligible toxicity and high biocompatible nature of the nanocarrier. The final BP-based nanoformulation (BP-DOX@PDA-TF) treated group exposed with NIR laser substantially reduced the cell viability with increase in concentration, demonstrating a dose-dependent cell toxicity effect, and exceptional photothermal conversion activity of BP-DOX@PDA-TF. Furthermore, plain DOX inhibited cell proliferation in a concentration-mediated pattern. However, no obvious toxicity effect was seen with NIR radiation in the free DOX group, demonstrating that the NIR exposure produced negligible harm to 4 T1 cells. Meantime, upon NIR laser irradiation at the same concentration, BP-DOX@PDA-TF exhibited considerably higher cellular toxicity compared to the rest groups. The results described can be quantitatively represented using IC50 values, evaluated using GraphPad Prism 8.0 software. The calculated IC50 values are given in the Table 1. As predicted, NIR irradiated BP-DOX@PDA-TF exhibited a significant reduction of IC50 from 3.19 to 0.451 μg/mL compared to non-irradiated groups. Additionally, the NIR-II light exposed groups showed a less IC50 (higher cytotoxicity) by BP-DOX@PDA-TF (0.4518) in comparison with DOX loaded BP (BP-DOX (4.16)) and free DOX (1.58) under identical environments. The results suggest an effective TF receptor-mediated active targeting by the TF ligand conjugated BP nanoformulation (Chen et al., 2020). Overall, BP-DOX@PDA-TF irradiated with NIR light could accomplish synergistic activity by combining chemotherapy with PTT, demonstrating greater efficacy than either treatment alone.

**Table 1:** IC-50 value for plain DOX, and BP-based nanoformulation for 4 T1 tumour cellular toxicity after 48 h.

Formulations	IC50 value (μg/mL)	
	Non NIR Irradiated	NIR Irradiated
BP	151.8	41.94
DOX	2.84	1.58
BP-DOX	7.16	4.16
BP-DOX@PDA-TF	3.19	0.45

#### 3.5.2 Cellular uptake evaluation

The cellular uptake characteristics of the prepared BP-based nanoformulation in comparison with free DOX was evaluated using flow cytometry in 4 T1 cells, a TNBC cell line. Cells treated with free DOX had greater fluorescence intensity than BP-based NPs. The reasoning behind this is that free DOX enters cells more quickly through passive diffusion, while BP-based nanoformulation usually enters cells through endocytosis. As we could see from the DOX release studies in different pH conditions, BP-DOX@PDA-TF release the DOX for a prolonged time in a controlled manner (Lee et al., 2008; Sun et al., 2010). The influence of NIR-II 808 nm irradiation towards cellular uptake of BP-based nanoformulation were carried out by exposing BP-DOX@PDA treated groups with laser. Consequently, the cellular uptake of BP-DOX@PDA substantially improved followed by NIR irradiation in comparison with non-irradiated groups (Fig. 6.F (i-iii)). This suggests that NIR may assist in DOX release by boosting the responsiveness and penetrability of tumour cells.

#### 3.5.3 Evaluation of TF receptor-mediated endocytosis

The transferrin receptor (TFR) is essential for cellular iron uptake as it binds to the protein-based ligand TF before internalizing it via the TF receptors. TF receptor 1 (TFR1) is located on the outer plasma membrane and is internalized into acidic endosomes within the cell through a clathrin/dynamin-dependent mechanism. This process enables iron transport into the cell, with TFR1 being recycled back to the cell surface. Notably, TFR1 levels are found to be 2–10 times higher in tumour cells than in normal cells (Bhatt et al., 2019; Kumari et al., 2018). TF-mediated cellular uptake of TF- conjugated BPNSs was evaluated by saturating 4 T1 cell TF receptors with a pretreatment of excess TF. The flow cytometry measurements of DOX intensity are shown in the Fig. 6.G (i-iii). Preincubation of 4 T1 cells with free TF and treatment with the BP-based nanoformulation, led to a notable reduction in fluorescence intensity in comparison with cells that were not pretreated with free TF. These findings clearly indicate that the TF-conjugated nanoformulation is internalized via TF receptor-mediated endocytosis.

#### 3.5.4 In vitro ROS estimation in tumour cells

Previous research has indicated that BP-based nanoformulation can generate intracellular ROS and cause cytotoxic effects. Combined with the observed cellular uptake and selective degradation of BP nanoformulations in cancer cells, we have strong evidence suggesting that the increased concentrations of phosphate anions in tumour cells lead to elevated ROS production. This, in turn, may result in the selective destruction of tumour cells by BP nanoformulation.

**Fig. 7:**
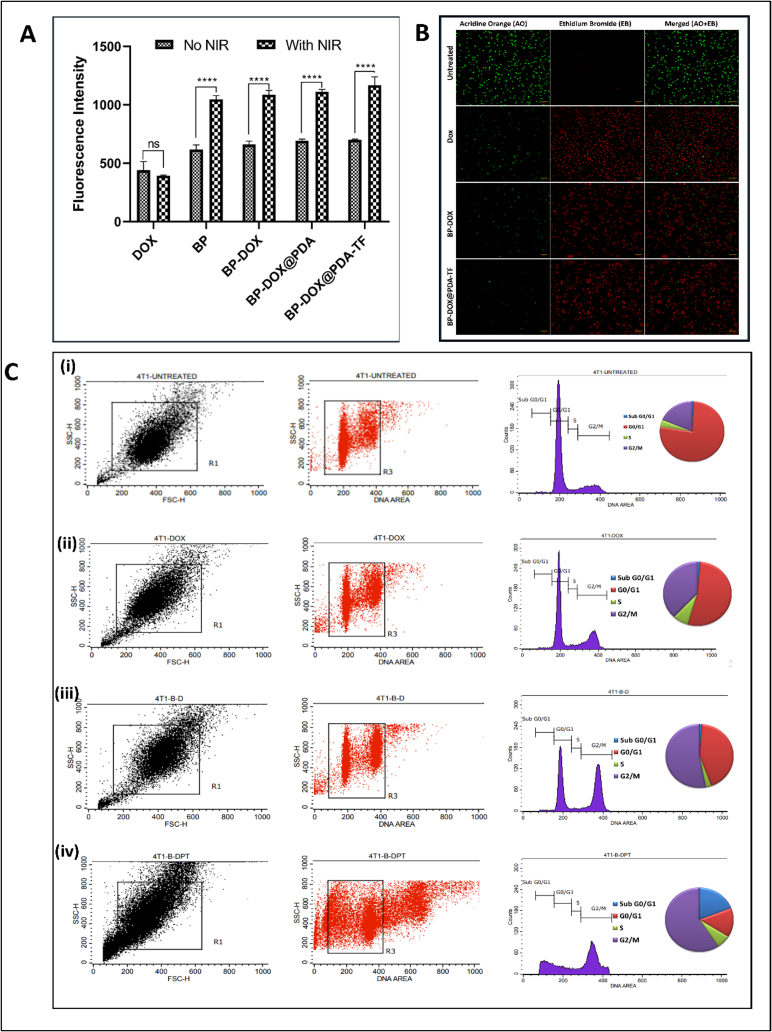
Intracellular ROS generation, apoptosis induction, and cell cycle arrest in 4 T1 cells treated with BP-based nanoformulations (A) In vitro ROS evaluation of Black Phosphorous-based nanoformulation using DCFH-DA and fluorescence microscopy in 4 T1 cells, (B) Apoptosis study of BP-based nanoformulation. Confocal microscopy images of Acridine orange/ethidium bromide (AO/EB) dual stained 4 T1 cells untreated, and treated with DOX, BP-DOX, and BP-DOX@PDA-TF. (C) Histogram plots illustrating cell cycle distribution arrested in different phases of 4 T1 cells in (i) Untreated cells, (ii) cells treated with DOX, (iii) cells treated with BP-DOX and (iv) cells treated with BP-DOX@PDA-TF.

To investigate this hypothesis, we looked at the influence of BP-based nanoformulation on ROS generation in 4 T1 cells. The photosensitive materials are capable of producing ROS in response to NIR irradiation, which leads to tumour ablation via apoptosis. DCFHDA assay kit was employed to assess the ROS production activity of BP-based nanoformulation in 4 T1 cells (Mu et al., 2017; Zhang et al., 2017). The results showed that the BP-based nanoformulation could generate a higher ROS compared to non-irradiated BP as illustrated in Fig. 7.A. Especially, the NIR light-exposed final formulation of BP (BP-DOX@PDA-TF) showed better ROS production. These outcomes were ascribed to enhanced cellular uptake and drug release triggered by ROS, leading to increased cytotoxicity and a lower IC50 value.

#### 3.5.5 Apoptosis study

Since apoptosis functions as the body's natural defense against aberrant cells, it is essential to cancer therapy. One of the main purposes of cancer therapy is to get cancer cells to undergo apoptosis. Apoptosis is a mechanism used by targeted medicines, radiation therapy, and chemotherapy to destroy cancer cells. These therapies try to stop cancer cells growth and shrink tumours by inducing apoptosis. Furthermore, inducing apoptosis aids in preventing the emergence of treatment resistance, a frequent obstacle in the management of cancer. Understanding apoptosis pathways better has allowed for the creation of targeted treatments that improve treatment outcomes by particularly enhancing apoptosis in cancer cells. Apoptosis-inducing medicines work even better when combined with other forms of treatment, providing a more all-encompassing strategy for controlling cancer and raising the chances of patient survival. As a result, apoptosis is a significant mechanism in the larger plan to combat cancer in addition to being a therapeutic target.

The impact of DOX, BP-DOX, and BP-DOX@PDA-TF on apoptosis induction was evaluated using EB/AO staining method. AO, an intercalating dye, attaches to DNA or RNA through electrostatic interactions and emits fluorescence in various shades, helping to visualize cell organelles. When AO binds to DNA, it fluoresces green, whereas EB fluoresces red. An undamaged nucleated cell emits green fluorescence, which indicates vitality. Early apoptotic cells likewise emit green fluorescence, but with chromatin condensation and nuclear disintegration. Late apoptotic cells are characterized by orange fluorescence and have shrunken, fragmented nuclei. Necrotic cells, on the other hand, appear enlarged like normal cells, with minimal chromatin condensation and exhibit orange to red fluorescence (Amaral et al., 2013). Previous reports indicated that 4 T1 cells treated with DOX exhibited both early and late apoptotic characteristics, with minimal or negligible necrosis (Yunita et al., 2020, p. 4). The Fig. 7.B. illustrates the morphological characteristics of 4 T1 tumour cells after 48 h of incubation with BP nanoformulations. (Akbarzadeh et al., 2020). An increase in the amount of necrotic and apoptotic cells in the cells treated with BP-DOX@PDA-TF was observed compared to plain DOX and BP-DOX, which further supports the findings observed in cell viability examination.

#### 3.5.6 Cell cycle analysis

Anticancer medicines are expected to modulate cell cycle progression. The different phases of cell cycle arrest generated by nanoformulations were investigated using flow cytometry (Monteiro et al., 2019; Yuan et al., 2016). Fig. 7.C depicts the histogram plots of cellular DNA content for various administration groups. The methodology employed for this analysis involved staining the DNA of collected cells with propidium iodide (PI) and RNase, followed incubation at RT for 15 mins. The 4 T1 cells were then evaluated using flow cytometry, focusing on the FL-2 channel to determine the DNA content and thereby measure the distribution of cells across the various stages of the cell cycle. This approach is well-established for evaluating cell cycle dynamics and has been shown to effectively highlight the cytotoxic effects of DOX, which include cell cycle arrest and subsequent apoptosis. The analysis of cell cycle progression in response to DOX treatment revealed significant alterations compared to untreated controls. In untreated cells, 76.81 % were in the G0/G1 phase, 3.72 % in the S phase, and 18.7 % in the G2/M phase. Following treatment with DOX and DOX loaded BPNSs, a marked change in the distribution of 4 T1 cells among the different phases of the cell cycle was detected.

Precisely, BP-DOX@PDA-TF resulted in only 5 % of cells remaining in the S phase, while a substantial quantity of 50 % were arrested in the G2/M phase. This indicates that BP-based nanoformulation could effectively disrupts normal cell cycle progression, promoting cell cycle arrest particularly at the S and G2/M phases, which is according to the recognized mechanism of inducing DNA damage and inhibiting cell proliferation by DOX.

The significant DNA damage induced by DOX likely leads to a higher number of cells being arrested in the G2/M phase. This phase is particularly susceptible to DOX-induced DNA damage. Arrest at the G2/M checkpoint can trigger apoptosis in cancer cells, intensifying the cytotoxic effects of DOX. These findings align with previous studies conducted on various cancer cell lines. Additionally, the data highlight a clear relationship between ROS and cell cycle processes, as DOX-loaded BPNS were found to increase intracellular ROS levels and disrupt cell cycle progression. As demonstrated in earlier research, elevated ROS levels can induce cell cycle arrest and apoptosis, especially in the context of DOX-induced cell death (Kim et al., 2009).

### 3.6 Preclinical animal studies

#### 3.6.1 In vivo pharmacokinetics

**Fig. 8:**
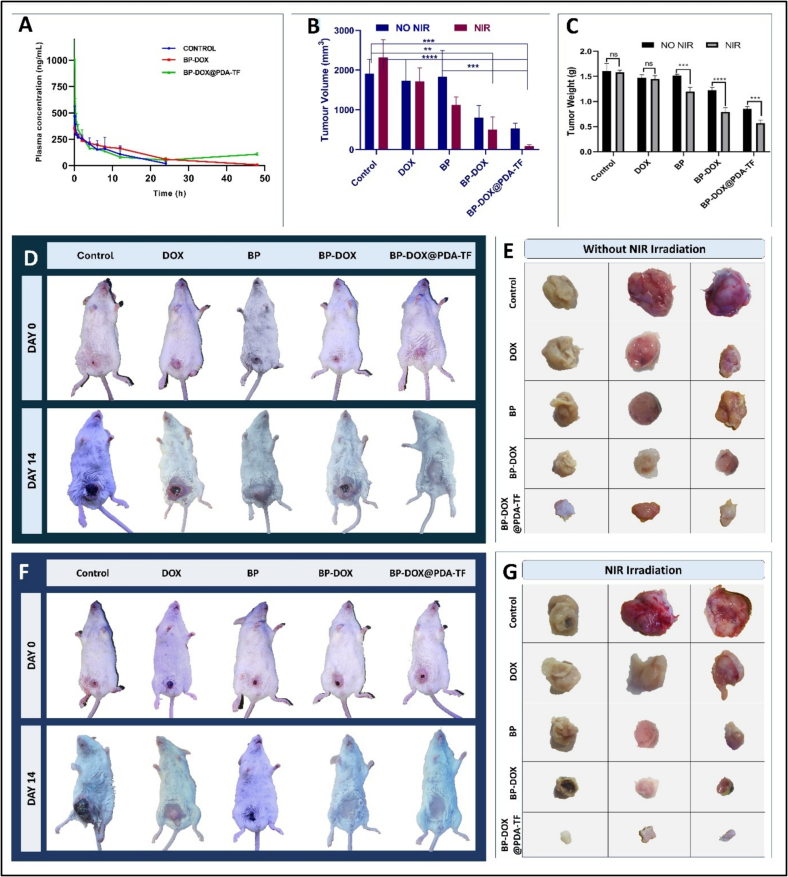
In vivo pharmacokinetics and antitumor efficacy of BP-based nanoformulations in BALB/c mice (A) In vivo pharmacokinetics of free DOX, BP-DOX and BP-DOX@PDA-TF after I.V. injection on BALB/c mice, indicating pharmacokinetic behaviour. (B) Tumour weight, and (C) tumour volume data after fourteen administrations of saline, DOX, BP, BP-DOX, and BP-DOX@PDA-TF demonstrating therapeutic efficacy of the formulations. Values are represented as Mean ± SD, *n* = 3. Image representing 4 T1 breast tumour bearing BALB/c mice administered with PBS, DOX, BP-DOX, and BP-DOX@PDA-TF at zero and fourteen days of treatment (D) without irradiation and (F) with NIR irradiation. In Vivo Antitumour Efficacy of Nanoformulation in 4 T1 breast cancer-Bearing BALB/c Mice: Tumour Photographs Post-Treatment (14 days) (E) without irradiation and (G) with NIR irradiation.

Anticancer drugs have numerous major drawbacks in terms of application, including a non-specificity, a reduced half-life, lesser drug localization in cancer tissues, and systemic toxicity. Drug-loaded nanocarriers may alter drug pharmacokinetic characteristics. This is particularly useful in keeping drugs from being digested or cleared rapidly (Ioele et al., 2022). The pharmacokinetic parameters of the plasma samples were evaluated using reverse phase HPLC by determining DOX concentration. As illustrated in Fig. 8.A, DOX stayed in the systemic circulation for a significantly longer duration in BP-DOX and BP-DOX@PDA-TF treated groups in comparison to free DOX. Non-compartmental analysis was used to calculate the pharmacokinetic behaviour of the formulations, which are represented in Table 2. AUC_0-t_ of BP-DOX and BP-DOX@PDA-TF was 1.5 (*p* < 0.001) and 1.91 (*p* < 0.0001) folds higher respectively compared to DOX. However, it was 1.22 folds higher (*p* < 0.01) for BP-DOX@PDA-TF compared to BP-DOX. This revealed that BP nanoformulations had higher bioavailability than free DOX. The PDA layer of the functionalized BP-based nanoformulation was able to protect DOX in the nanosheet and control its release to a significant degree compared to BP-DOX during the flow in blood. Comparable findings were observed with the AUC_0-∞_, wherein the BPNS-based nanoformulation exhibited higher values (*p* < 0.001) in comparison with free DOX group. The maximum concentration (C_max_) values of indicated that plasma concentration of DOX is improved once it is loaded and functionalized into BPNSs. This improvement is attributable to the regulated DOX release and its increased stability. The earliest possible sampling time was 5 min, which corresponded to the T_max_ for all groups. Several prior investigations on DOX showed a similar T_max_ value of 5 min (Zeng et al., 2019; Zhong et al., 2020).

**Table 2:** The important pharmacokinetic values of free DOX and BP-based nanoformulation.

Parameters	DOX	BP-DOX	BP-DOX@PDA-TF
AUC_(0-t)_ (ng/mL*h)	2976.15 ± 1191.277	4654.04 ± 354.16 ***	5709.899 ± 638.69 ****#
AUC_(0-∞)_ (ng/mL*h)	3168.98 ± 1086.246	4781.377 ± 424.48 **	7826.70 ± 1123.81 ****^####^
C_max_ (ng/mL)	469.73 ± 93.78	354.68 ± 26.14	1016.04 ± 265.22
T _max_ (h)	0.083 ± 0.00	0.083 ± 0.00	0.083 ± 0.00
t_1/2_ (h)	5.47 ± 2.15	8.20 ± 3.44	16.64 ± 5.08
Ke (h^−1^)	0.141 ± 0.05	0.09 ± 0.04	0.045 ± 0.016

All the values are given as Mean ± SD, *n* = 3; ** *p* < 0.01 in comparison with free DOX; ***p < 0.001 in comparison with free DOX; ****p < 0.0001 in comparison with free DOX; ^#^*p* < 0.05 in comparison with BP-DOX; ^####^*p* < 0.0001 in comparison with BP-DOX.

The free DOX treated groups showed a short half-life of 5.47 ± 2.15 h, indicating its rapid elimination from the systemic circulation. The TF conjugated PDA functionalized BP-based nanoformulation exhibited an improved half-life of 16.64 ± 5.08 h. These results showed that loading of DOX onto BPNSs caused an improvement in the prolongation of t_1/2_. This longer half-life can help to maintain therapeutic DOX concentrations for a longer period of time, possibly boosting its efficacy in treating the breast cancer. The elimination rate constant (K_e_) of BP nanoformulation was significantly lower than that of DOX, indicating more efficient circulation in the blood stream. The formulation's prolonged circulation period may reduce the unfavourable effects of DOX to the major organs while increasing drug localization in tumour tissues. The effective particle diameter of BP-DOX@PDA-TF of about 256 nm permits for effective DOX localization towards cancer cells by evading reticuloendothelial system or rapid removal from circulation (Liu et al., 2021). These results indicate that the PDA layer may significantly improve circulation time, which is the mechanism underlying improved cancer targeting. As a result of greater systemic circulation and improved pharmacokinetic parameters, BP-based nanoformulation are projected to exhibit more therapeutic activity for cancer treatment.

#### 3.6.2 Pharmacodynamic studies

Encouraged by the excellent tumour cell-targeting capability of BP-DOX@PDA-TF during in vitro evaluation studies, the in vivo antitumour therapeutic activity of the BP-based nanoformulation was further carried out in 4 T1-breast tumour bearing BALB/c mice by i.v. injection of nanomaterials. For the simultaneous observation of photothermal activity of the formulation, NIR thermal imaging camera was employed to evaluate the thermal variations in the tumour and to record thermographic maps. Upon NIR laser exposure, the temperature of the tumour region of animal group treated with BP-DOX@PDA-TF rose from 30 °C to a maximum of 64 °C within 5 min, which is enough for tumour thermal ablation. However, the animal groups administered with saline, BP, DOX and BP-DOX exhibited relatively slighter temperature elevation. This significant variation can be described by the difference in cellular uptake of nanoformulations by tumour cells.

The vivo anticancer activity of different formulations given to the animal groups was further confirmed by tumour size in terms of volume and weight after tumour collection was measured. As illustrated in Fig. 8.B, tumour volumes in the Control and Saline + NIR Laser irradiated groups increased rapidly throughout the observation period, demonstrating that NIR irradiation as such is insufficient to obstruct breast tumour development under the same investigational conditions. In contrast, tumour volumes in the BPNSs + NIR Laser and BP-DOX + NIR light laser groups gradually decreased followed by NIR laser (808 nm) irradiation. After 14 days of observation, the tumours in both treatment groups had been effectively eradicated. Tumours treated with BPNS and NIR 808 nm laser were more efficiently reduced in size than those in untreated animals (Fig. 8.C), indicating that the BP nanostructures have a strong antitumour effect.

As illustrated in Fig. 8.D and F both DOX and BP-DOX treatments failed to efficiently suppress the breast tumour growth, mostly due to poor uptake by 4 T1 cells. Effective tumour growth inhibition was observed in BP-DOX@PDA-TF plus 808 NIR laser (1.0 W/cm^2^ power density) treated animals. In this tumour group, the tumour inhibitory activity was almost uniform in all three mice, with a significant tumour weight reduction compared with saline administered group. The excellent anticancer effectiveness of BP-DOX@PDA-TF combined with photothermal therapy can be ascribed to the excellent tumour uptake of the nanoformulation, which leads to a significant generation of ROS within the tumour and intense photothermal effects. Among all treatments, BP-DOX@PDA-TF in combination with NIR 808 nm light irradiation demonstrated the greatest efficacy in cancer ablation, with only small tumour tissues detected. Furthermore, the average tumour weight (Fig. 8E and G) indicated an increased antitumour activity of the BP-mediated PDT-PTT. The findings from our in vitro and in vivo experiments align well with previously reported studies highlighting the potential of BP-based nanocarriers in targeted drug delivery and combined cancer therapy. Notably, in the work by Xu et al., BPQDs camouflaged with a platelet-osteosarcoma hybrid membrane (BPQDs-DOX@OPM) demonstrated prolonged circulation, enhanced tumour targeting, and superior antitumor efficacy compared to monotherapy (Xu et al., 2023). Another recent study presented a pH-responsive and photothermal-triggered drug delivery system utilizing PEGylated BPNSs loaded with indocyanine green (ICG) for fluorescence imaging-guided breast cancer therapy (Pan et al., 2021). The BPNSs demonstrated excellent biocompatibility, high drug-loading capacity, and effective photothermal conversion under NIR irradiation, leading to enhanced tumour targeting and therapeutic efficacy.

##### 3.6.2.1 In vivo thermal images

**Fig. 9:**
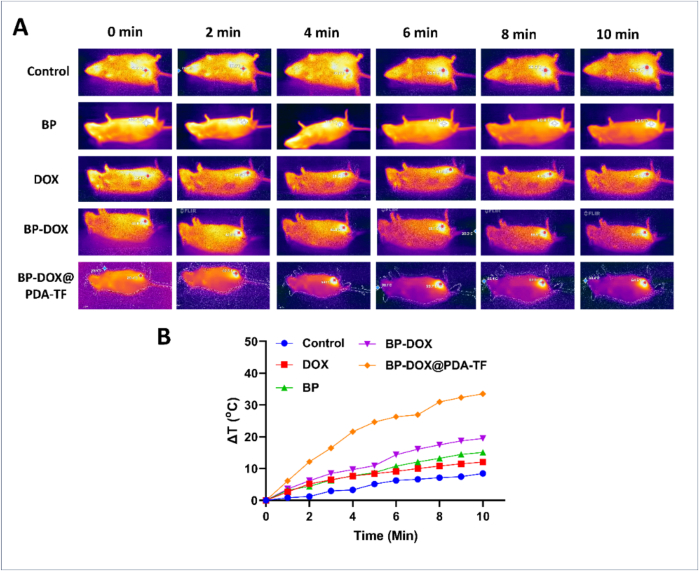
Photothermal response of BP-based nanoformulations in breast cancer-bearing mice (A) Thermal imaging of breast cancer mice model; post-intravenous administration of saline, DOX, BP, BP-DOX, and BP-DOX@PDA-TF, with 808 nm light exposure (1.0 W/cm^2^) and (B) Corresponding temperature profiles over time, showing the photothermal conversion efficiency of each treatment group.

The photothermal images of the prepared nanoformulation was further evaluated using thermal imaging camera. The animals were exposed with NIR 808 nm (1.0 W/cm^2^) light for 10 min at the tumour site after 24 h of administration of saline, DOX, BP, BP-DOX and BP-DOX@PDA-TF. The temperature changes at the tumour site were measured at every 15 s and recorded. A substantial increase in the tumour tissue temperature upon NIR laser exposure could be ascribed due to the notable photothermal characteristics of PDA coat and BPNSs. The temperature of the tumour treated with BP-DOX was 57.4 °C and it was 63.6 °C for BP-DOX@PDA-TF administered animals. This rise in temperature is adequately sufficient for efficient tumour killing. The localized tumour tissue temperature of BP-DOX and BP-DOX@PDA-TF formulation treated groups altered evidently compared to plain BP and DOX treated groups. Conversely, following irradiation under the same settings, saline administered group did not show a significant temperature increase, indicating that the tumour cells are not affected. The PDA-coated, TF-conjugated BP-based nanoformulation demonstrated significant photothermal activity and prolonged circulation, with the ability to target tumour tissues in a pH-responsive manner. Quantified variations in tumour temperature are shown in Fig. 9.

The observed results are in agreement with previous work by Gao et al., who reported pH-responsive dual drug-loaded poly(2-ethyl-2-oxazoline) modified BP nanosheets for chemo/photothermal therapy, and highlighted the capability of BP-based systems for in vivo photothermal imaging and controlled heat generation under NIR light exposure (Gao et al., 2019). In their study, thermal imaging was effectively used to visualize and confirm photothermal behaviour at the tumour site, consistent with our results. The correlation between thermal imaging and treatment efficacy further underscores the diagnostic and therapeutic value of BPNSs in biomedical applications.

##### 3.6.2.2 Histopathological analysis in tumour tissues and other organs

**Fig. 10:**
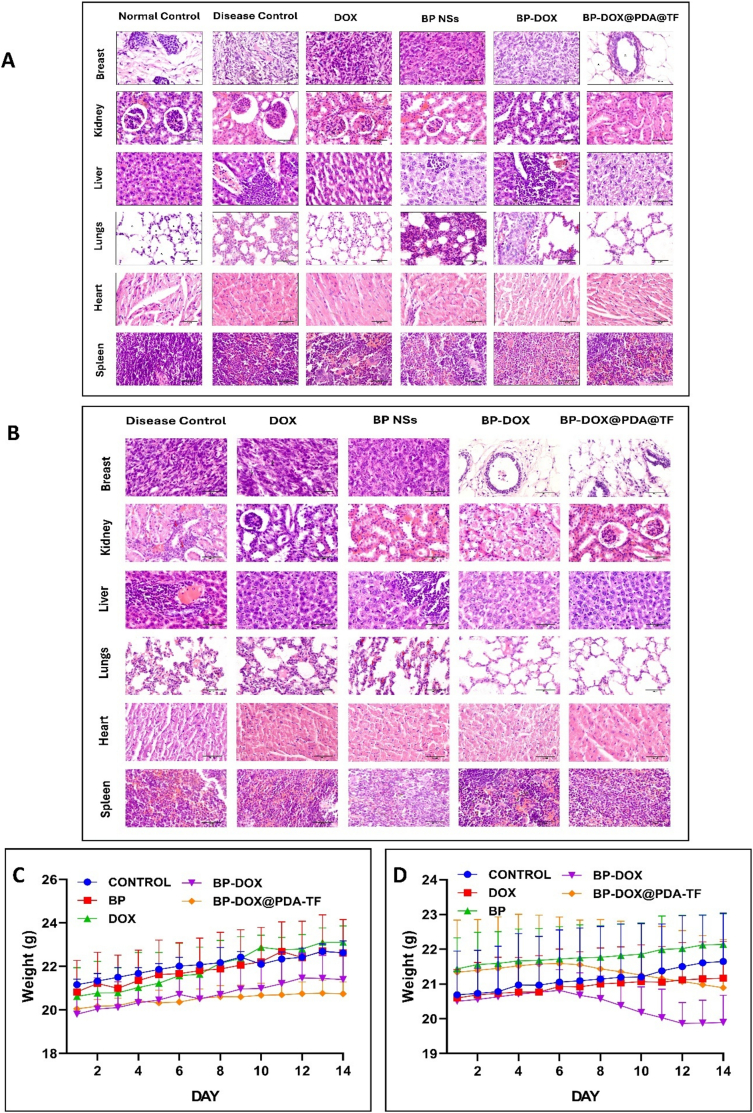
Histopathological analysis of major organs after administration with saline, DOX, BP, BP-DOX, and BP-DOX@PDA-TF after 14 days of treatment (A) without irradiation, and (B) with NIR irradiation. Change in body weight from zero to fourteen days of treatment (C) Treatment group with no laser, and (D) NIR laser treated groups.

Histological evaluation of the H&*E*-stained tissues from the BALB/c mice was performed. The tissue section from the normal saline-administered group reveals numerous tumour cells infiltrating the fibrous stroma in clustered formations. These tumour cells exhibit hyperplasia with pronounced malignant features, including cellular and nuclear pleomorphism, characterized by substantial difference in the size and shape of cells and nuclei. The nuclei are hyperchromatic, indicating a darker stain due to excessive chromatin, and there is an increased nuclear-to-cytoplasmic ratio with prominent nucleoli. Necrotic areas with loss of cellular and nuclear details are evident. Mitotic figures are present, indicating active cell division. Additionally, acute inflammatory infiltration is observed in certain regions. Notably, the invasion of tumour cells into the muscle tissue is also apparent. Most of the areas of tissue section treated with plain DOX shows stroma without tumour cells and necrosed areas. Few tumour cells showing hyperchromatic nuclei is seen in one area. The stroma shows severe infiltraion of acute inflammatory cells. The muscle area is free of tumour invasion with the presence of severe acute inflammatory infiltration. The BPNSs administered tumour sections demonstrated many tumour cells infiltrating the fibrous stroma scattered and in the form of clusters. Areas of necrosis with loss of cellular and nuclear details, as well as mitotic figures, are observed. Tumour cell invasion into the muscle tissue is also evident. The tissue section of BP-DOX treated animals shows many tumour cells infiltrating the fibrous stroma scattered and in the form of clusters. Areas of necrosis showing loss of cellular and nuclear details are seen. Some areas show mild reduction in nuclear pleomorphism and mitotic figures. The BP-DOX@PDA-TF treated tissue section shows mammary gland tissue and lymph node. The mammary gland area shows glandular alveoli and ducts scattered in the adipose tissue. There are no tumour cells in the tissue section with no metastasis (*Table S1; Supplementary Information*). Additionally, HE staining reveals a substantial increase in tumour necrosis and a notable reduction in residual tumour cells in the NP photothermal treatment group (Fig. 10.A and B). This indicates that the NPs exhibit the most pronounced tumour clearance effect following photothermal therapy.

Histopathological investigation revealed that malignancies had metastasized to the liver and kidneys in the untreated positive control group. While the breast tumours were completely ablated the treatment groups, there were substantial variations in liver pathology among regimens. Mice treated with DOX showed severe hypertrophy and liver congestion, but the NP treatment groups showed only moderate to mild hypertrophy. These data indicate that DOX and its formulations can prevent liver metastasis to varied degrees, with BP-DOX@PDA-TF treatment being the most effective, followed by BP-DOX, then pure DOX. No abnormalities were found in the kidneys of any therapy group. These findings show that the BP-based nanoformulation increased the efficiency of DOX and effectively inhibited tumour spread in vivo.

##### 3.6.2.3 Change in body weight

Toxic side effects often result in weight loss. During treatment, no decrease in BALB/c mouse body weight was detected (Fig. 10.C and D), indicating that the BP-based nanoformulation has no noticeable detrimental side effects (such as animal death or abnormality in body weight), significant biocompatibility and negligible in vivo toxicity.

##### 3.6.2.4 Blood parameters estimation biochemical parameters estimation

Standard blood chemistry parameters such as RBCs, white blood cells (WBCs), platelets (PLTs), mean corpuscular hemoglobin (MCH), and mean corpuscular volume (MCV) were used to assess hematological toxicity. As shown in *Table S2 and S3; Supplementary Information*, the levels of WBCs, PLTs, and other parameters remained within acceptable limits, indicating that the BP-based nanoformulation treatment did not cause severe hematotoxicity.

**Fig. 11:**
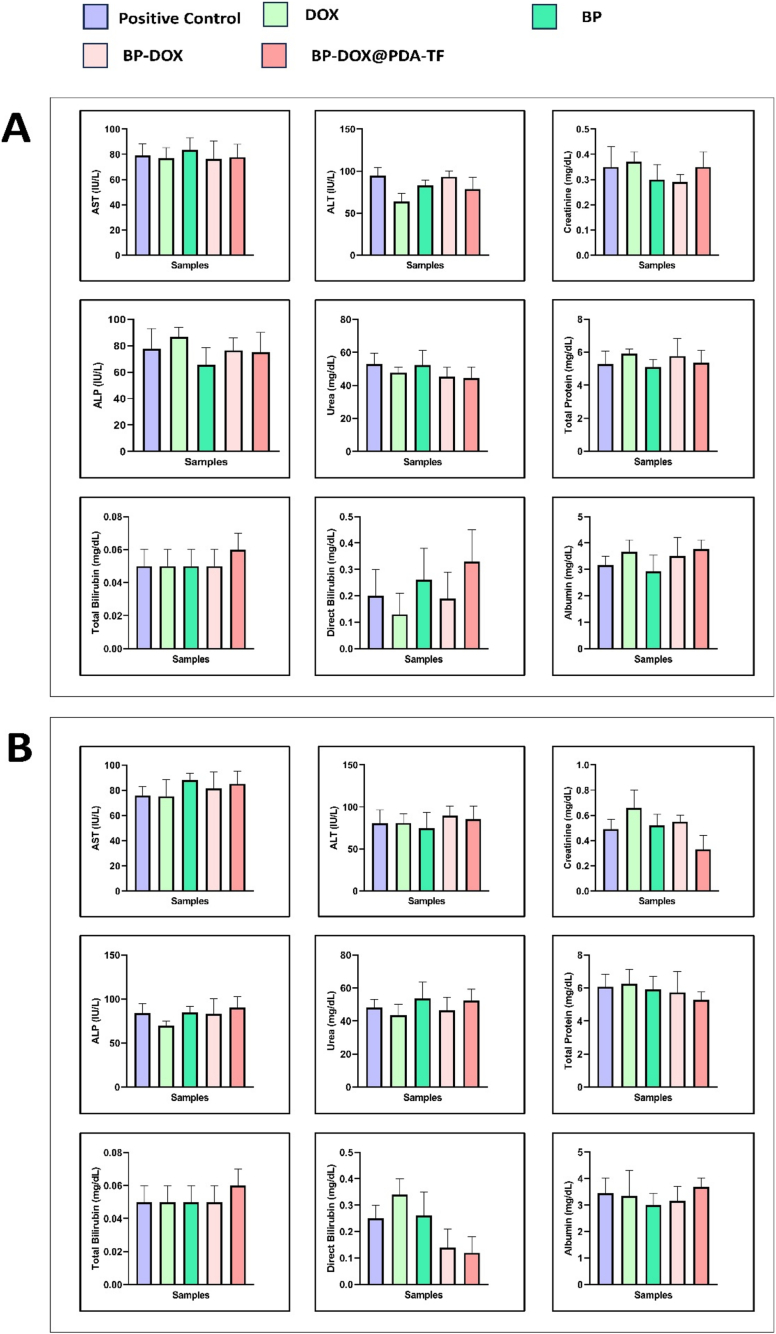
Evaluation of biochemical parameters in rats treated with BP-based nanoformulations with and without NIR irradiation. Different biochemical parameters of NIR irradiated rats administered with saline, DOX, BP, BP-DOX, and BP-DOX@PDA-TF after 14 days (A) without irradiation, and (B) with NIR irradiation to assess systemic toxicity and organ function following photothermal therapy.

We also measured important indicators of hepatic and renal function, including urea, aminotransferase (ALT), aspartate aminotransferase (AST), and alkaline phosphatase (ALP). Fig. 11.A and B shows that there were no substantial changes in these markers between the treatment and control groups, demonstrating that the BP-DOX@PDA-TF treatment is safe for the liver and kidneys. The blood biochemical analysis revealed no substantial differences between the BP-DOX@PDA-TF and saline-treated groups, demonstrating excellent biocompatibility of the prepatred BP-based nanoformulation.

##### 3.6.2.5 IL-6 estimation by ELISA analysis

Uncontrolled cell proliferation has been associated with tumour growth, immune evasion, and an increased metastatic risk. These changes are frequently linked to modifications in cytokine synthesis. IL-6 is a cytokine that controls inflammation and metastatic tumour activity. According to studies, people with breast cancer had increased IL-6 level. Furthermore, higher IL-6 levels are related with tumour growth, progression, and metastasis. It leads to an unspecific up-regulation of the cytokine in most cancers associated with inflammation (Waldner et al., 2012). Previous research has demonstrated that animals with breast tumours and increased IL-6 levels develop spontaneous metastases in the lungs and liver (Oh et al., 2013).

**Fig. 12:**
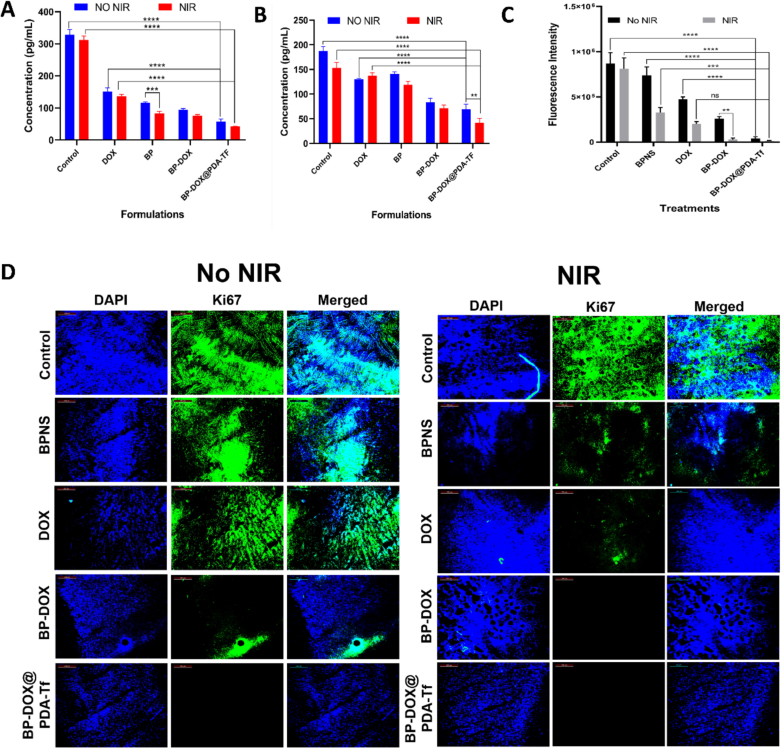
Inflammatory cytokine levels and tumour proliferation marker analysis following treatment with BP-based nanoformulations. Estimation of (A) IL-6 levels and (B) TNF-α levels of animal groups by ELISA treated with saline, DOX, BP, BP-DOX, and BP-DOX@PDA-TF with and without NIR laser irradiation. (C) Ki-67 quantification by measuring the fluorescence intensity of the graph. (D) Representative confocal images of cryo-sectioned tumour tissues from BALB/c mice: DAPI-stained nuclei (left panel) and Ki-67 green fluorescence (middle panel), showing proliferation status across different treatment groups. (For interpretation of the references to colour in this figure legend, the reader is referred to the web version of this article.)

The obtained results showed that IL-6 levels were substantially less in animal groups exposed to NIR irradiation, specifically on BPNSs nanoformulation treated animals that is BP-DOX (75.93 ± 3.70 pg/mL) and BP-DOX@PDA-TF (42.16 ± 0.43 pg/mL) compared to non-irradiated groups of is BP-DOX (94.72 ± 3.73 pg/mL) and BP-DOX@PDA-TF (57.58 ± 8.31 pg/mL) (Fig. 12.A.). Control groups of both irradiated and non-irradiated groups exhibited no significant difference which confirms the photothermal activity of BPNSs. These results show the significant part of IL-6 in breast tumour metastasis. Salgado et al. found that high IL-6 blood levels were linked to decreased survival rates in advanced breast cancer patients. At later phases, elevated levels may also contribute to cancer progression (Salgado et al., 2003).

##### 3.6.2.6 TNF-α estimation by ELISA analysis

TNF-alpha has critical functions in breast cancer because it regulates inflammation, tumour cell proliferation, survival, metastasis, and immune response. It promotes cancer development by creating a pro-inflammatory milieu, increasing cancer cell proliferation and survival, and enabling metastasis via extracellular matrix breakdown. TNF-alpha also encourages the immune system into attacking cancer cells. TNF-alpha levels are elevated in breast cancer patients, indicating a poor prognosis, underscoring its importance as a biomarker for diagnosis and therapy outcome. Understanding TNF-alpha's various roles is critical for designing tailored treatments and improving patient outcomes. Clinical studies suggest that TNF-α may have prognostic value. TNF-α is present in both the tumour site and the circulation of cancer patients, suggesting it plays a role in both primary tumour growth and metastasis. However, more research into the molecular pathways by which this cytokine influences breast cancer initiation, progression, and invasion is needed to fully comprehend its potential in cancer care. The impact of TNF-α on breast cancer development has primarily been studied in vitro with cell lines originating from the disease. Significantly decreased expression of TNF-α was also observed in laser treated BP-based nanoformulation as shown in Fig. 12.B. In summary, BP-DOX@PDA-TF showed great potential in reducing tumour-related inflammation through the suppression of TNF-α.

##### 3.6.2.7 Ki-67 estimation

Ki67 is a nuclear antigen found in cells undergoing active division. Several studies (Fasching et al., 2011) have shown a significant association between the immunological response of Ki67 and the cell cycle. It is present in the cell cycle's G1, S, G2, and M phases but not in the G0 phase. Ki67 expression is limited during late G1 and early S phases and then gradually increases during the S phase, with a significant rise observed in the later part of the cell cycle. Furthermore, the measurement of Ki67 levels during neoadjuvant chemotherapy can be used as an indicator to predict the likelihood of achieving complete elimination of cancerous tissue in patients with breast cancer. A high Ki67 level following neoadjuvant treatment suggests a poor prognosis. Therefore, Ki67 is considered a crucial indicator for detecting the proliferation of cancer cells. An example of this is a clinical trial (Tan et al., 2014) that validated the dependability of Ki67 as a prognosticator for pathological complete response followed by neoadjuvant chemotherapy with DOX. The Ki67 labelling index was a more precise determinant of tumour response to treatment with chemotherapeutics in HR-negative breast cancer. In a separate neoadjuvant investigation (Fasching et al., 2011) with 552 individuals with breast cancer, the inclusion of Ki67 considerably improved the capacity to forecast the response to treatment in triple-negative and luminal tumours. Therefore, we examined the role of Ki67 as a prominent indicator of tumour growth in assessing the efficacy of DOX-loaded BPNS formulations in treating breast cancer. The figures display tumour portions corresponding to various treatment groups. The fluorescence intensity of Ki-67 staining followed a decreasing trend: Control > BPNSs > DOX > BP-DOX > BP-DOX@PDA-TF (Fig. 12.C.). Among the groups, BP-DOX@PDA-TF exhibited the lowest green fluorescence, indicating a significant reduction in tumour cell proliferation (Fig. 12.D.). Further, upon exposure to NIR radiation, there was a noticeable drop in the fluorescence intensity compared to the respective groups with no NIR irradiation. A noteworthy observation was that the BPNS-based formulations (BP-DOX and BP-DOX@PDA-TF) receiving NIR irradiation showed a great reduction in intensity, indicating the photosensitivity of BPNS. As demonstrated in the in vitro studies, BPNS, a photosensitizer, absorbs the NIR radiation and leads to photothermal therapy of the tumours. Similar observations were also reported earlier (Jia et al., 2022). The substantial reduction in Ki-67 intensity in the NIR-irradiated BP-DOX@PDA-TF group highlights the enhanced antiproliferative effect of the combined chemo–photothermal–photodynamic therapy.

##### 3.6.2.8 ROS detection assay

**Fig. 13:**
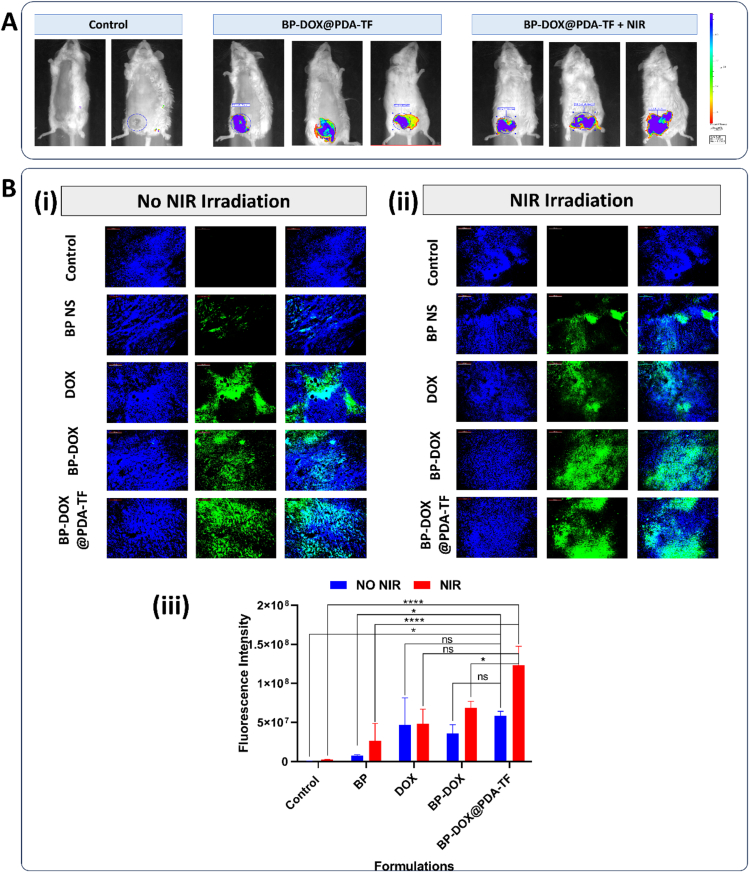
In vivo reactive oxygen species (ROS) generation by BP-based nanoformulations with and without NIR irradiation (A) Live fluorescence imaging of BALB/c injected with the saline and BP-based nanoformulation with and without NIR irradiation which was intratumourally administered with DCFH-DA, and (B) Fluorescence images of DCFH-DA administered tumour tissue slices (excitation/emission: 488/520 nm) of (i) Group received no NIR irradiation, (ii) Group received NIR irradiation, and (iii) Total body fluorescent intensity analysis.

The collected tumour sections were evaluated for ROS production in the tumours using live imaging. A significant rise in green fluorescence was detected in BP-DOX@PDA-TF compared to BP-DOX, BPNSs, DOX and Normal Saline treated groups. The formulation, BP-DOX@PDA-TF, produced ROS in a significant quantity compared to BP-DOX, BPNSs, DOX and normal saline treated groups resulted in apoptosis and tumour ablation causing enhanced anticancer activity compared to other formulations. The fluorescence intensity, which reflects ROS generation, was measured in mice 30 min after DCFH-DA injection, with the results presented in Fig. 13. The data demonstrated a statistically significant increase in ROS production in mice administered with BP-DOX@PDA-TF in comparison with other treated groups. The results demonstrates that the ROS generated by BPNSs under external irradiation can release from BP-DOX into DOX.

Upon NIR laser irradiation, BP-DOX@PDA-TF not only converts near-infrared laser into localized hyperthermia for PTT but also generates a substantial amount of reactive ROS for photodynamic therapy (PDT), demonstrating the effective phototherapeutic capabilities of BPNSs. The DOX-loaded BPNSs releases DOX as a result of ROS generation, allowing it to enter the nucleus and exert its chemotherapeutic effects. As a result, the combinational chemo-phototherapy proves to be significantly more effective than phototherapy as such. Furthermore, the observed cellular uptake and selective rapid degradation of BPNSs within cancer cells support the notion that elevated phosphate anion levels in these cells lead to increased ROS production, which can selectively induce cell death via BPNSs. To explore this hypothesis, we assessed the impact of the BP-based nanoformulation on ROS production in cancer cells. The activated BPNSs, when exposed to NIR laser of 808 nm, produces ROS capable of destroying tumour cells via mechanisms such as apoptosis or necrosis.

##### 3.6.2.9 TUNEL assay

**Fig. 14:**
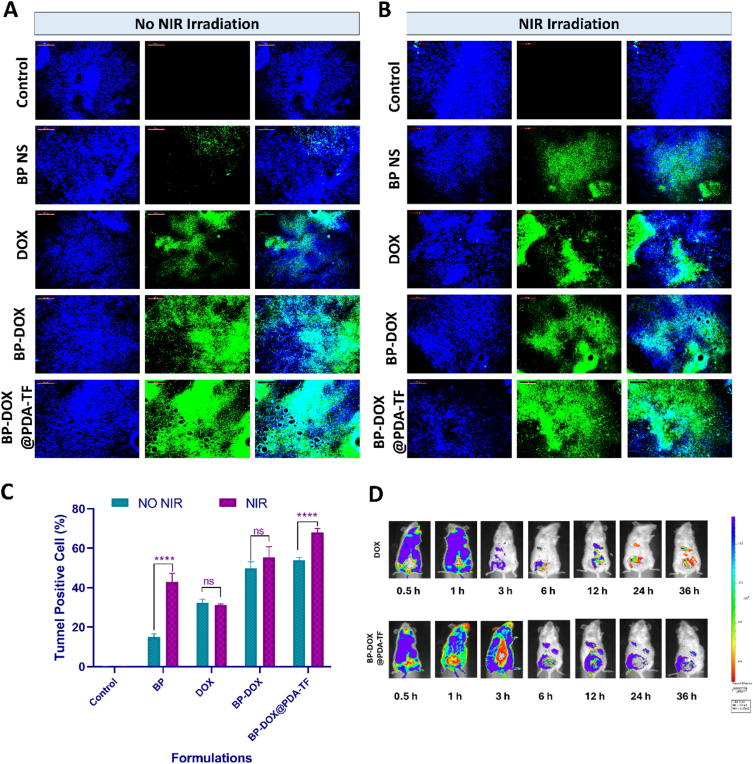
TUNEL assay results of tumour sections. Tissue sections stained with DAPI in the left side, while the middle panel displays apoptotic nuclei labeled with green fluorescence (A) without and (B) with NIR irradiation. (C) Quantification of TUNEL-positive cells was conducted as shown. (D) Biodistribution studies in BALB/c mice administered with DOX and BP-DOX@PDA-TF at 0.5–36 h post-injection using live optical fluorescence imaging. (For interpretation of the references to colour in this figure legend, the reader is referred to the web version of this article.)

The antitumour efficacy of photothermal therapy (PTT) against 4 T1 tumours was assessed using the TUNEL immunohistological assay, which exclusively detects apoptosis in tumour tissues. This assay revealed apoptotic cells within the tumour tissues. As depicted in the Fig. 14. A-C, tumours in the PBS and PBS + NIR groups showed negligible apoptosis, underscoring the biosafety of the NIR laser. Because it lacks tumour-targeting capabilities, BP-DOX cannot accumulate selectively in tumour tissue, resulting in a DOX concentration that is insufficient to trigger apoptosis in the BP-DOX group. However, with the combination of the BP-based photothermal effect, the BP-DOX + NIR group exhibited significant apoptosis following illumination. BP-DOX@PDA-TF, which has excellent tumour-targeting properties, can specifically accumulate at the tumour site, but without irradiation, the drugs remain confined in the nanosheets, unable to induce cell apoptosis. Upon irradiation, the drugs are released and can perform their pharmaceutical functions. These results explains that BP-DOX@PDA-TF is an efficient antitumour agent capable of inducing significant photo-induced cell killing.

#### 3.6.3 Biodistribution study

The 4 T1-breast tumour bearing BALB/c mice was intravenously injected with free DOX, and BP-DOX@PDA-TF NPs to examine the in vivo distribution of BP-based nanoformulation. The in vivo fluorescence signals of DOX were measured at 0.5, 1, 3, 6, 12, 24, and 36 h showed that 30 min followed by i.v. administration, both DOX and BP nanoformulation-administered animals exhibited an intense fluorescence signal at the tumour region. However, the fluorescence signal in the BP-based nanoformulation-administered animals gradually increased up to 12 h post-injection, whereas DOX-administered animals showed a significantly reduced signal. This indicates that the BP-DOX@PDA-TF may progressively accumulate at cancer tissue region. The animal groups treated with BP-DOX@PDA-TF has exhibited an intense fluorescence signal after 24 h of administration, demonstrating that the BP-DOX@PDA-TF NPs have improved targeting and retention ability as given in Fig. 14.D. The fluorescence intensity of the BP-DOX@PDA-TF NPs administered animals showed much better distribution than in the DOX-treated animals, demonstrating an efficient accumulation in the tumour location.

## 4 Conclusions

In this research, BP-based nanoformulation was prepared for tumour-specific synergistic chemo/photothermal therapy of breast cancer. It showed efficient DOX encapsulation with higher in vitro cell uptake and improved cytotoxicity. The nanoformulation was photo-responsive in nature with pH/NIR-responsive DOX release characteristics. Cell line studies using 4 T1 cells revealed potential toxicity of the formulation towards NIR irradiated groups with enhanced ligand-mediated cellular uptake. PDA layer significantly improves circulation time, which is the mechanism underlying increased tumour targeting. Pharmacodynamic studies on 4 T1 tumour bearing BALB/c mice revealed excellent antitumour effectiveness of BP-DOX@PDA-TF combined with photothermal therapy (PTT) can be ascribed to the high tumour uptake of the nanoformulation, which leads to a significant generation of ROS within the tumour and intense photothermal effects. In summary, the BP-based nanoformulation could improve the breast tumour localization and prolonged DOX circulation. The BP-based nanoformulation has extensive application in synergistic cancer treatment and breast cancer theranostics.

## CRediT authorship contribution statement

**Soji Soman:** Writing – original draft, Methodology, Formal analysis, Data curation. **Sanjay Kulkarni:** Writing – original draft, Methodology, Formal analysis, Data curation. **Jeena John:** Methodology, Formal analysis, Data curation. **Milan Paul:** Methodology, Formal analysis, Data curation. **Krishnadas Nandakumar:** Writing – review & editing, Validation, Resources, Data curation. **Swati Biswas:** Writing – review & editing, Validation, Methodology, Data curation. **Sajan D. George:** Writing – review & editing, Validation, Methodology, Data curation. **Srinivas Mutalik:** Writing – review & editing, Validation, Supervision, Resources, Project administration, Data curation, Conceptualization.

## Declaration of competing interest

The authors declare no competing interests.

## Data Availability

Data will be made available on request.

## Appendix A Supplementary data

Supplementary material Image 1
