# Supplementary material for: Transferrin conjugated pH/NIR-responsive black phosphorus nanoplatform: A novel multimodal approach for breast cancer theranostics
Source: Int J Pharm X. 2025 Jul 22;10:100364. doi: 10.1016/j.ijpx.2025.100364 (PMC12356476; doi:10.1016/j.ijpx.2025.100364)
Supplement: Supplementary file 1 — Supplementary material [file mmc1.docx]

**Transferrin conjugated pH/NIR-responsive black phosphorus nanoplatform: A multimodal approach to breast cancer theranostics**

Soji Soman ^a^, Sanjay Kulkarni ^a^, Jeena John ^b^, Milan Paul ^c^, Krishnadas Nandakumar ^b^, Swati Biswas ^c^, Sajan D George ^d^, Srinivas Mutalik ^a*^

^a^ Department of Pharmaceutics, Manipal College of Pharmaceutical Sciences, Manipal Academy of Higher Education, Manipal 576104, Karnataka, India

^b^ Department of Pharmacology, Manipal College of Pharmaceutical Sciences, Manipal Academy of Higher Education, Manipal 576104, Karnataka, India

^c^ Department of Pharmacy, Birla Institute of Technology & Science-Pilani, Hyderabad Campus, Medchal, Hyderabad 500078, Telangana State, India

^d^ Department of Atomic and Molecular Physics, Manipal Academy of Higher Education, Manipal 576104, Karnataka, India

***Corresponding Author**

Dr. Srinivas Mutalik

Professor and Principal

Manipal College of Pharmaceutical Sciences

Manipal Academy of Higher Education, Manipal 576104

Karnataka, India

Email: ss.mutalik@manipal.edu

**Table S1. Histopathology results of tumour tissues treated with different formulations**

| **Irradiation** | **Rat no and section** | **Tumour cells** | **Necrosed area** | **Malignant features like nuclear pleomorphism, hyperchromatism** | **inflammation** | **Muscle invasion** | **Mitotic count/10HPF** |
| --- | --- | --- | --- | --- | --- | --- | --- |
| NIR non-irradiated | Positive Control | +++ | + | +++ | ++ | +++ | 30 |
|  | DOX | + | - | ++ | +++ | - | 4 |
|  | BP | +++ | +++ | +++ | +++ | ++/ +++ | 39 |
|  | BP-DOX | +++ | ++ | ++(some areas)  +++ | +++ | ++/+++ | 19 |
|  | BP-DOX@PDA-TF | - | - | - | + | - | - |
| NIR irradiated | Positive Control | +++ | +++ | +++ | +++ | ++ | 41 |
|  | DOX | +++ | +++ | +++ | - | - | 23 |
|  | BP | ++/+++ | + | ++/+++ | +++ | + | 12 |
|  | BP-DOX | - | +++ | - | - | - | - |
|  | BP-DOX@PDA-TF | - | + | - | +++ | - | - |

- Nil, + few, ++ Many +++ Abundant

**Table S2. Haematological parameters of 4T1 tumour bearing BALB/c mice administered with saline, DOX, BP, BP-DOX, and BP-DOX@PDA-TF after 14 days of treatment**

| **Parameters** | | **Normal Range** | | **Control** | | **BP** | | **DOX** | | **BP-DOX** | | **BP- DOX@P DA-TF** | |
| --- | --- | --- | --- | --- | --- | --- | --- | --- | --- | --- | --- | --- | --- |
| WBC  (10^3^/µL) | 1.5-10 | | 9±1.65 | | 9.97±1.1 | | 9.73±1.5 | | 9.83±1.27 | | 9.68±0.15 | |  |
| RBC  (×10^6^/µL) | 7-9.5 | | 8.41±1.07 | | 8.83±0.7 | | 9.39±1.2 | | 9.69±0.64 | | 7.89±1.46 | |  |
| HCT (%) | | 42-52 | | 42.14±1.77 | | 44.13±2.22 | | 42.69±4.36 | | 43.67±2.31 | | 42.6±2.16 | |
| HCT (%) | | 42-52 | | 42.14±1.77 | | 44.13±2.22 | | 42.69±4.36 | | 43.67±2.31 | | 42.6±2.16 | |
| HGB (g/dL) | | 13-17 | | 14.07±1.52 | | 15.97±1.66 | | 14.57±0.42 | | 15.02±0.61 | | 14.43±1.5  6 | |
| MCV (fL) | | 50-55 | | 50.92±3.29 | | 51.8±2.93 | | 52.77±4.81 | | 51.72±6.23 | | 52.77±3.1 | |
| MCH (pg) | | 16-18 | | 17.92±0.63 | | 15.75±1.56 | | 16.27±1.64 | | 16.27±0.76 | | 16.37±0.2  5 | |
| MCHC  (g/dL) | | 31-35 | | 35.68±3.31 | | 34.47±2.97 | | 35.23±1.06 | | 33.73±2.36 | | 35.73±0.5  4 | |
| RDW‐CV  (%) | | 11-15 | | 14.03±0.21 | | 14.27±2.21 | | 13.77±0.38 | | 14.27±0.49 | | 14.7±0.96 | |
| RDW‐SD  (fL) | | 35-55 | | 36.87±1.98 | | 36.47±3.94 | | 38.17±3.09 | | 41.3±6.15 | | 38.73±2.0  3 | |
| PLT  (×10^3^/µL) | | 668-  1543 | | 954±46.51 | | 893.67±116.07 | | 940±75.19 | | 958.67±62.1  3 | | 921.33±8  4.06 | |
| MPV (fL) | | 6-10 | | 5±0.1 | | 6.7±1.55 | | 6.37±1.21 | | 7.1±1.39 | | 7.23±1.16 | |
| PDW (fL) | | 10-20 | | 15.1±1.31 | | 15.5±0.96 | | 14.77±2.06 | | 14.6±1.06 | | 14.87±0.2  2 | |

**Table S3. Haematological parameters of 4T1 tumour bearing BALB/c mice administered with saline, DOX, BP, BP-DOX, and BP-DOX@PDA-TF after 14 days of treatment and NIR Irradiation**

| **Parameters** | **Normal Range** | **Control** | **BP** | **DOX** | **BP-DOX** | **BP- DOX@PDA**  **-TF** |
| --- | --- | --- | --- | --- | --- | --- |
| WBC (10^3^/µL) | 1.5-10 | 9.93±0.55 | 8.51±0.45 | 8.77±0.81 | 9.17±0.45 | 9.67±0.91 |
| RBC (×10^6^/µL) | 7-9.5 | 9.4±0.46 | 7.07±0.81 | 7.8±1.31 | 9.53±0.83 | 9.72±0.98 |
| HCT (%) | 42-52 | 43.29±4.0  9 | 41.87±2.19 | 46.76±4.31 | 43.57±4.05 | 43.57±4.45 |
| HGB (g/dL) | 13-17 | 13.4±1.11 | 11.6±0.61 | 13.88±1.28 | 13.73±0.68 | 13.53±10 |
| MCV (fL) | 50-55 | 49.91±2.60 | 54.93±4.80 | 50.93±3.10 | 50.83±4.20 | 51.17±2.87 |
| MCH (pg) | 16-18 | 17.86±0.93 | 17.5±0.92 | 16.35±1.51 | 16.5±0.62 | 17.2±0.30 |
| MCHC  (g/dL) | 31-35 | 34.14±2.31 | 34.6±2.17 | 32.51±30 | 31.8±2.07 | 31.13±4.09 |
| RDW‐CV (%) | 11-15 | 15.11±0.79 | 14.99±0.78 | 13.61±1.26 | 12.6±1.85 | 13.5±1.18 |
| RDW‐SD (fL) | 35-55 | 39.7±2.07 | 36.49±4.97 | 35.71±3.29 | 39±3.94 | 35.57±4.29 |
| PLT (×10^3^/µL) | 668-  1543 | 1039.06±54.10 | 788.98±41.27 | 924.2±85.27 | 1000±26.15 | 876±80.62 |
| MPV (fL) | 6-10 | 6.23±0.32 | 7.74±0.40 | 6.39±0.59 | 6.67±0.71 | 6.57±0.81 |
| PDW (fL) | 10-20 | 17.45±0.91 | 15.18±0.79 | 14.34±1.32 | 13.33±1.63 | 11.53±1.97 |
